# Investigation of the Phenolic Profile and Chemotaxonomical Screening of Twelve *Salix* Species During Growing Season

**DOI:** 10.3390/plants15111712

**Published:** 2026-06-01

**Authors:** Leonie Kayser, Thomas Olaf Gruber, Gregor Aas, Guido Jürgenliemk

**Affiliations:** 1 Department for Pharmaceutical Biology, University of Regensburg, Universitätsstraße 31, 93053 Regensburg, Germany; leonie.kayser@chemie.uni-regensburg.de (L.K.); thomas.gruber@chemie.uni-regensburg.de (T.O.G.); 2 Ecological-Botanical Gardens Bayreuth, University of Bayreuth, Universitätsstraße 30, 95447 Bayreuth, Germany; gregor.aas@uni-bayreuth.de

**Keywords:** *Salix*, willow, phenolic compounds, chemotaxonomical screening, phytochemical screening, correlation, interspecific variability, interannual variability, interseasonal variability, intersexual variability

## Abstract

The present study describes the phenolic profile of the leaves of 12 different willow species by UPLC^®^-RP18-PDA in terms of variability between species, years, harvest month, and among sexes. The analyzed compound classes include salicylic alcohol derivatives (SADs), caffeic acid derivatives (CADs), coumaryl alcohol glucosides (CAGs), flavan-3-ols, flavanones, flavanonols, flavones, and flavonols. Furthermore, the *Salix* species were chemotaxonomically classified on this basis, and correlations between the constituent classes were analyzed. The investigations indicate that the phenolic spectrum is highly species-specific but reveals no sex-specific variation. The flavan-3-ol content varied substantially among species, ranging from 2.1–36 mg/g DW and *S. bicolor* was determined to be a species of particular phytochemical interest, as it contained high concentrations of flavanonols (13 mg/g DM) and flavones (24 mg/g DW). Furthermore, at the end of the growth period, the secondary metabolite content was significantly higher and the compound classes CADs and flavan-3-ols were found to be significantly influenced by the harvest year. Chemotaxonomical classification revealed the separation of *S. bicolor*, *S. purpurea*, and *S. caprea* from the other species when compared with the generally accepted classification of the genus. This study contributes to a more comprehensive phytochemical characterization of willow species, which may support the development of willow extracts.

## 1. Introduction

The genus *Salix* L. comprises about 500 species with highly diverse growth forms, ranging from low-growing shrubs to tall trees, including numerous hybrids [1,2]. Most species are dioecious, although monoecious forms, such as the hybrid S. × sepulcralis, have been reported [3,4]. The inflorescences are spikes, commonly known as catkins in willows [5] and the leaf morphology varies considerably among species [3].

*Salix* species are distributed globally and occupy diverse ecological niches, from lowland habitats to alpine environments [6,7,8]. Their ecological success is based on characteristics such as a high tolerance to frost and flooding, rapid regeneration, and the ability to colonize disturbed habitats [6]. Furthermore, willows act as ecosystem engineers by influencing the availability of resources and promoting a wide range of ecological interactions with pollinators, herbivores and microorganisms [6,9,10,11,12].

Phenolic compounds play a central role in plant physiology by contributing to antioxidant defense, UV protection, and resistance against pathogens and herbivores, whilst also responding flexibly to biotic and abiotic environmental factors [11,12,13,14]. Their biosynthesis is strongly influenced by environmental conditions, suggesting that both species identity and habitat conditions have an influence on phenolic profiles.

Among these compounds, salicylic alcohol derivatives represent the most comprehensively studied compound class in *Salix* due to their pharmacological significance [5,15]. Flavan-3-ols, including monomeric compounds such as catechin and oligomeric compounds such as proanthocyanidins, also represent a main constituent class and occur in high concentrations in both the bark and the leaves [5]. Like SADs, they are considered to have herbivore-repellent properties [16,17,18]. Other flavonoids, including flavanones, flavanonols, flavonols, and flavones, are widespread in various parts of the plant and include bioactive compounds such as naringenin, quercetin or luteolin [5]. In addition, phenylpropanoic acids, such as p-coumaric acid, caffeic acid and their derivatives, represent an important group of phenolic compounds that are frequently found in leaves and bark [12,19,20,21,22]. The functions of flavonoids and phenylpropanoic acids in the plant are to provide protection against UV radiation, oxidative stress and herbivores [23,24,25].

Willow bark (Salicis cortex) is used for rheumatic diseases, fever, headaches, and low back pain [26]. Although the European Pharmacopoeia lists no specific willow species for pharmaceutical use, a minimum SAD concentration of 1.5%, expressed as salicin is required [27]. Though the anti-inflammatory, antipyretic, and analgetic effects are generally attributed to salicin as the main active constituent, other substance classes, such as flavonoids and phenolic acids, play a role in the context of synergism as well [15,28,29,30,31]. Beyond these traditional applications, various *Salix* species and their constituents exhibit a broad spectrum of biological activities, including antioxidant, anticarcinogenic, cytotoxic, antidiabetic, antimicrobial, anti-obesity, neuroprotective and hepatoprotective properties [5].

Research focused on the phytochemical characterization of different species within *Salix* has primarily concentrated on salicylic alcohol derivatives present in the bark [32,33,34,35,36,37,38,39,40,41]. In contrast, willow leaves are often disposed of as waste after bark collection. However, willow leaves also contain a wide variety of phenolic compounds, including SADs, CADs, flavonoids, and condensed tannins, and could represent a valuable resource [35,36,38,42,43].

Previous research has consistently demonstrated pronounced interspecific variability in phenolic compound composition within *Salix* [32,33,36,37,38,40,44]. For example, species such as *S. fragilis* have been reported to contain high levels of CADs [43], whereas other species, including *S. cinerea*, *S. caprea*, and *S. viminalis*, show considerably lower concentrations [45]. Similarly, the flavan-3-ol content varies markedly among species, with *S. caprea* and *S. cinerea* typically exhibiting high levels, while *S. fragilis* contains comparatively low amounts [43,46]. Flavonoid composition is likewise species-dependent, with certain species, such as *S. purpurea*, showing elevated concentrations of specific compounds like naringenin, rutin, and quercetin [43,47]. These findings indicate that phenolic profiles are strongly species-specific and may provide valuable chemotaxonomic information. In addition to interspecific differences, the phenolic composition is also influenced by harvest time and environmental factors. Seasonal fluctuations have been reported to substantially influence metabolite concentrations, with CADs and CAGs showing maxima at the beginning of the growing season and flavonoids towards the end [39,40]. However, these patterns are not consistent across species [40]. Interannual variability remains less understood, with conflicting findings reported in the literature, because it is challenging to distinguish the effects of harvest years from climatic variations [48,49,50,51,52,53]. The influence of sex on phenolic compound composition is still debated, with some studies reporting higher concentrations of defensive compounds in female individuals [54,55], whereas others found no significant differences [56,57,58,59]. In summary, the phenolic content is influenced by the species, environmental conditions, and physiological response.

Despite numerous classification approaches, including morphological [60,61], phylogenetic [1,62], and phytochemical [20,37,40,46,47] exploration, the taxonomy of *Salix* remains complex due to frequent hybridization without displaying significant morphological differences [53].

While various investigations have described the variability of phenolic compounds in *Salix*, considerably less attention has been focused on the relationships between different phenolic compound classes and their biosynthetic pathways. Many of these compounds are connected via shared metabolic pathways, such as phenylpropanoid and flavonoid biosynthesis, where intermediates like CADs serve as precursors for downstream products including flavanones, flavonols, and flavan-3-ols. Consequently, fluctuations in the concentration of a particular compound class may reflect changes in enzymatic activity that occur in response to environmental changes. Investigating correlations between phenolic compound classes may therefore provide indirect insights into potential precursor–product relationships and regulatory mechanisms during the growth period. However, such relationships have rarely been explored in *Salix*, particularly across multiple species and phenolic classes [14,63,64,65,66].

Based on these observations, this study examines the hypothesis regarding whether the phenolic compound classes exhibit species-specific patterns, how their concentrations are significantly influenced by the harvest time, whether sex influences the content of the phenolic constituent classes, how they can be classified on the basis of their phenolic compound profile, and whether correlations between the compound classes might reflect underlying biosynthetic relationships.

This investigation builds on a previous study investigating the SAD content in *Salix* species [38]. While the earlier study focused on the intra- and interspecific, as well as inter-seasonal, interannual, and intersexual variability of the SAD content, the present analysis examines this variability within other relevant phenolic compound classes, such as CADs, CAGs, flavan-3-ols, flavanones, flavanonols, flavonols, and flavones, of the same species and individuals. Quantitative analysis was performed using a UPLC^®^-RP18-PDA method, and statistical evaluation was conducted using linear regression models. In addition, based on these and the previously published data [38], the species were classified chemotaxonomically by using principal component analysis (PCA) and hierarchical clustering. Furthermore, investigations on correlations between the substance classes were conducted to better understand their biosynthetic relationships and seasonal dynamics, using Pearson correlation coefficients. The methods for sampling and extraction followed the procedures described previously, with the modifications listed below.

To enhance the quality of willow-based phytopharmaceuticals, it is essential to obtain plant materials rich in bioactive compounds. This requires further exploration and detailed characterization of the phenolic profiles of willow species.

## 2. Results

### 2.1. Interspecific Variability

Table 1, Table 2 and Table 3 indicate the mean values with standard deviation (SD) for each species based on the entire dataset and the regression coefficients of the linear mixed model for species-specific effects. Furthermore, Table 4 and Table 5 display the overall effect (χ^2^) of the species variable on the content of the different compound classes, indicating that species had a highly significant effect on all analyzed compound classes as a result of the type II ANOVA.

The CAD content was highest in *S. bicolor*, *S. fragilis*, and *S. hastata*, whereas *S. bicolor* differed from most other species, except for *S. fragilis* and *S. hastata* (Table 1). However, *S. daphnoides* contained the lowest CAD levels. The highest overall content, concerning a specific individual, was observed in *S. bicolor* in May 2019 (57.2 ± 0.6 mg/g DW). CAD content varied substantially among species, ranging from 2.3 to 15 mg/g DW with a highly significant species effect (χ^2^ = 2015.263, *p* < 0.001) (Table 4).

The *S. bicolor* individual also contained the highest detected CAG content and *S. purpurea* the lowest. Except for *S. aurita*, *S. caesia*, and *S. lapponum*, the CAG content detected in *S. bicolor* was significantly higher than the content of most of the other species (Table 1). In September of 2019, the individual with the highest content (1.73 ± 0.10 mg/g DW) belonged to the species *S. aurita*. CAG content showed interspecific variation, spanning a range from 0.02 to 0.5 mg/g DW, and was strongly influenced by the species variable (χ^2^ = 368.879, *p* < 0.001) (Table 4).

For the flavan-3-ols, *S. caprea* represented the species with the highest amounts, followed by *S. aurita*, *S. cinerea*, and *S.* × *sepulcralis. S. caprea* also differed significantly from the other species, except for *S. aurita* and *S.* × *sepulcralis* (Table 1). Furthermore, *S. caesia* showed the lowest flavan-3-ol levels. In terms of a particular individual, *S. caprea* had the highest total content in August 2018 (71 ± 3 mg/g DW). Flavan-3-ol content exhibited substantial interspecific variation, ranging from 2.1 to 36 mg/g DW, with species identified as a major determinant (χ^2^ = 2443.197, *p* < 0.001) (Table 4).

The highest flavanone content was detected in *S. purpurea*, followed by *S. daphnoides* (Table 2). Here too, the content of *S. purpurea* was significantly higher than the others, apart from *S. aurita*, *S. caesia*, *S. daphnoides*, and *S. hastata*. In contrast, the lowest flavanone level was found in *S. cinerea*. The highest detected content, related to a specific individual, was in August 2018 in the species *S. purpurea* with 58.48 ± 0.28 mg/g DW. Flavanone concentration varied considerably among species, in a range from 2.2 to 29 mg/g DW with a highly significant species effect (χ^2^ = 1311.313, *p* < 0.001) (Table 5).

*S. bicolor* also represented by far the species with the highest flavanonol content, which was significant (Table 2). Any other species contained less than 1 mg/g DW, except for *S. lapponum* and *S. purpurea*. In July 2019, *S. bicolor* contained the highest overall concentration (44.1 ± 0.4 mg/g DW) for a specific individual. Flavanonol content was not as strongly influenced by the species variable as the other compound classes (χ^2^ = 80.713, *p* < 0.001), as shown in Table 5, displaying an interspecific content variation ranging from 0.011 to 13 mg/g DW.

As with the previous substance class, *S. bicolor* contained the most flavones, followed by *S. purpurea* and *S. fragilis* (Table 3). Though *S. bicolor* differed not significantly from most of the other species, except for *S. caprea*, *S. cinerea*, and *S. lapponum*. In terms of a particular individual, in the species *S. purpurea* the highest detected content of 83 ± 4 mg/g DW was found in August 2019. Flavone content exhibited interspecific variation, ranging from 1.3 to 24 mg/g DW, with species identified as an important factor (χ^2^ = 1366.758, *p* < 0.001) (Table 5).

The flavonol content was highest in *S. bicolor* and *S. lapponum* and was significantly higher than in the other species, which contained less than 1 mg/g DW flavonols (Table 3). The lowest levels were detected in *S. fragilis* and the highest overall content, concerning a specific individual, was found in *S. hastata* in August 2019 (6.7 ± 1.2 mg/g DW). Flavonol content showed a highly significant species effect (χ^2^ = 285.123, *p* < 0.001), with interspecific differences in content ranging from 0.05 to 5 mg/g DW (Table 5).

### 2.2. Interannual Variability

The effect of year on the content of the different phenolic compound classes varied, with both significant and non-significant patterns observed. The year 2018 was used as the reference, and effect estimates therefore reflect differences relative to 2018 based on log-transformed concentration data in mg/g DW (Table 4 and Table 5).

The CAD and flavan-3-ol contents are significantly influenced by the years in which the plants were harvested, as shown in Table 4. In the subsequent year, the flavan-3-ol concentration decreased (R^2^ = −0.18 ± 0.06, *p* < 0.01) while the CAD level increased (R^2^ = 0.11 ± 0.03, *p* < 0.01).

For the flavanone (R^2^ = 0.052 ± 0.027), flavone (R^2^ = 0.10 ± 0.06), and flavonol (R^2^ = 0.001 ± 0.020) contents, the effect of the year was positive but not statistically significant, indicating no differences relative to 2018 (Table 5).

The levels of flavanonol (R^2^ = −0.006 ± 0.028) and CAG (R^2^ = −0.005 ± 0.012) displayed the opposite trend, though it was similarly not statistically significant (Table 4 and Table 5). This indicates that, for the remaining phenolic compound classes, the harvest year has no influence on the content.

### 2.3. Interseasonal Variability

Statistical analysis revealed that the harvest time had a significant influence on the concentration of each substance class, except for flavanonols (Table 4 and Table 5). The effect of the month variable on the content of the different phenolic compound classes is displayed as log-transformed concentration estimates in mg/g DW with May as reference.

Each substance class showed their maximum content towards the end of the growth period, except for CAD (Table 4 and Table 5, Figure S1A–G). The peak months for CAD were May through June, apart from *S. purpurea* (August) and *S. viminalis* (September) (Figure S1A). The minima often occurred between July and September, except for *S. × sepulcralis* (May) and *S. purpurea* (June).

The maximum CAG content was recorded between August and September, with the lowest levels predominantly in May and never in September (Figure S1B).

Half of the species showed their highest flavan-3-ol contents at the beginning of the growth period (May–June) and the other half at the end (July–September) (Figure S1C). Despite this, apart from *S. fragilis* (September), the majority of the species presented their minimum in May or July.

With the exception of *S. caesia* (June), *S. purpurea* (July), and *S. lapponum* (August), the highest levels of flavanones were found in May or September, whereas the lowest levels varied widely among the species (Figure S1D).

The flavanonol concentration maxima were detected between July and September, apart from *S. aurita*, *S. caprea*, and *S. viminalis*, which showed their maxima in May. Though not very evident, there was a tendency for the lowest levels to occur between July and September (Figure S1E).

The highest flavone content was found in June for about half of the species, and July through September for the other half (Figure S1F). However, it was also highly variable in terms of the minima. The tendency was more towards the beginning of the growth period (May–June).

In terms of maxima, the flavonol content was distributed similarly to the flavones and the minima also varied considerably but were never detected in September (Figure S1G).

### 2.4. Intersexual Variability

The effect of sex on the different phenolic compound classes was generally weak and statistically not significant (Table 4 and Table 5). Females were used as the reference, and effect estimates therefore reflect differences relative to female individuals based on log-transformed concentration data in mg/g DW.

For the substance class CAD, there was only a small and not significant effect explained by sex (R^2^ = 0.02 ± 0.09) (Table 4). This indicates that CAD concentrations in male individuals did not substantially deviate from those observed in females. Similarly, CAG also showed a negligible association with sex, suggesting no meaningful differences between male and female individuals (Table 4).

For flavan-3-ols, the effect of sex was slightly higher (R^2^ = 0.07 ± 0.11), but remained low and statistically not significant (Table 4). In contrast, flavanones exhibited the highest R^2^ value among all compound classes (R^2^ = 0.20 ± 0.13), suggesting a more pronounced, yet not statistically significant, deviation of male individuals from female flavanone concentrations (Table 5).

For the remaining flavonoid classes, the effect of sex on the content was minimal and negative (Table 5). Flavanonols showed a negative R^2^ value (R^2^ = −0.11 ± 0.11), indicating that no consistent differences relative to females were detectable. Similarly, flavones were largely unaffected by sex. This was also the case for flavonols, which also yielded comparable results, indicating no significant deviation from female reference levels.

Overall, sex has no significant influence on the content of the phenolic compound classes examined, even though females seem to have a slight tendency to exhibit higher levels of flavanonols, flavones, and flavonols. Whereas, for the CAD, CAG, flavan-3-ol, and flavanone contents, the opposite trend was recognizable. However, no statistically significant differences between male and female individuals were detected.

### 2.5. Phytochemical Classification

**Figure 1:**
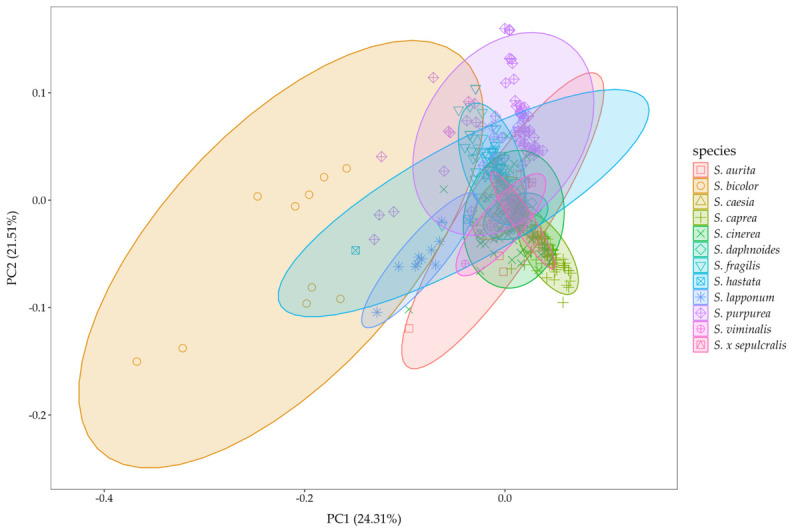
Principal component analysis (PCA) score plot of the investigated *Salix* species based on the contents of SADs, CADs, CAGs, flavan-3-ols, flavanones, flavanonols, flavones, and flavonols. Each point represents one biological sample (mean of triplicate measurements), color-coded by species. The ellipses indicate the distribution and grouping of samples within each species.

This *Salix* screening was conducted to classify the examined species on a phytochemical basis and to compare or supplement this with existing morphological [1], phylogenetic [2,25], and phytochemical [22,23,24] classifications. Therefore, a PCA was performed with the mean values of each technical triplicate according to the SAD, CAD, CAG, flavan-3-ol, flavanone, flavanonol, flavone, and flavonol contents (Figure 1).

**Figure 2:**
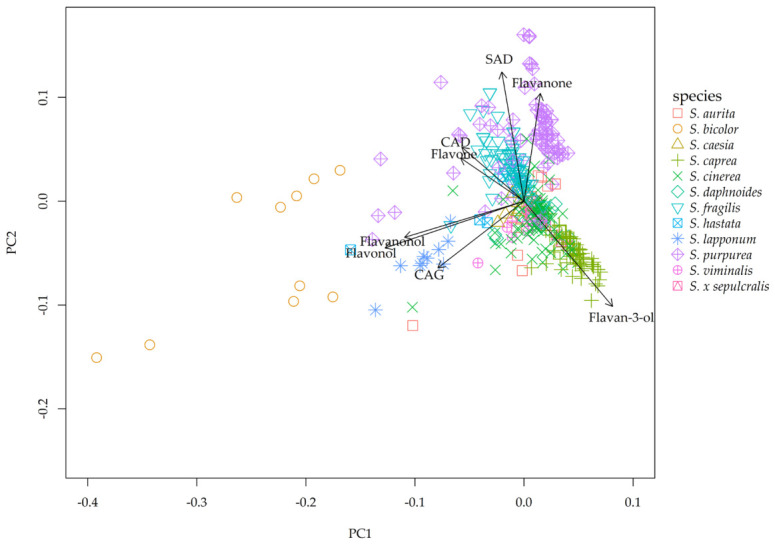
Loading plot of principal component analysis (PCA) of phenolic compound contents in the twelve analyzed species showing the distribution of samples and the contribution of phenolic compound classes to the first two principal components (PC1 and PC2). Samples are color-coded according to species, while arrows represent the loadings of the respective compound classes. The orientation and length of the vectors indicate the direction and strength of variable contributions, accordingly.

PC1 and PC2 explained 45.82% of the total variance of the data set. The substance class flavonol (−0.580) contributed most to PC1 (24.31%), followed by flavanonol (−0.498) and flavan-3-ol (0.369) (Figure 2). PC2 accounted for 21.51% of the total variance and was characterized by positive loadings of SAD (0.566) and flavanone (0.473), and by negative loadings of flavan-3-ol (−0.460). A high value for PC1 indicates high contents of flavan-3-ol and low levels of flavonols and flavanonols. Similar to PC2, high values present high SAD and flavanone concentrations, and low contents for flavan-3-ols.

Overall, the PCA loading plot revealed relationships between the investigated compound classes (Figure 2). Flavan-3-ols were oriented in the opposite direction to most other phenolic classes, indicating a strong negative association, particularly with CAD and flavone. In contrast, SADs and flavanones showed a similar orientation, suggesting a positive relationship between these compound classes.

In general, two distinct clusters were recognizable (Figure 1). *S. bicolor* formed a distinct group, clearly separated from all other species, which were largely grouped in a second cluster. Within this second cluster, *S. purpurea* and *S. caprea* were more clearly differentiated compared with the remaining species.

*S. bicolor* was strongly differentiated from the other species due to negative values for PC1 and PC2. In contrast, *S. purpurea* represented the opposite, with mostly positive values for PC1 and PC2, whereas *S. caprea* displayed an intermediate position with positive values for PC1 and slightly negative values for PC2. All of the other species arranged in between exhibited high positive values for PC1 and were scattered along the PC2 axis.

**Figure 3:**
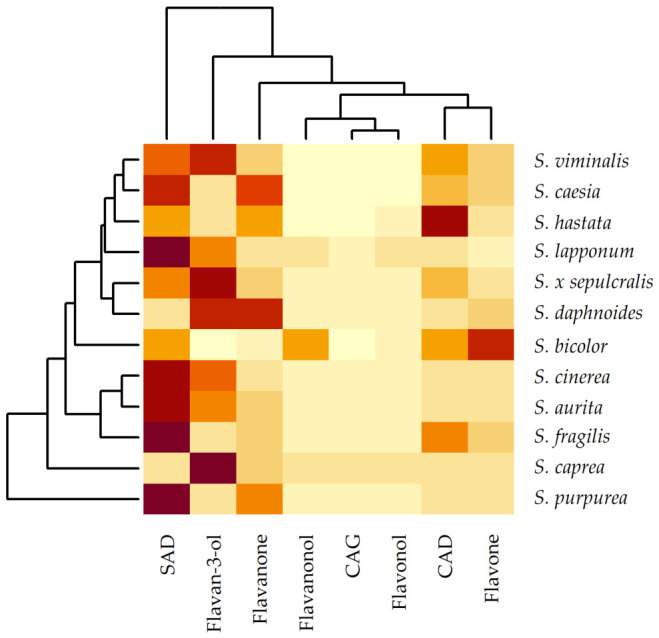
Hierarchical clustering based on the mean values of the phenolic substance classes and on their abundance patterns across samples. Rows represent compound classes (SAD, CAD, CAG, flavan-3-ol, flavanone, flavanonol, flavone, and flavonol), and columns correspond to the species. Color intensity reflects the mean content of each compound class in mg/g DW, with darker shades indicating higher contents and lighter shades indicating lower contents. Dendrograms indicate the similarity between compound classes and samples based on hierarchical clustering using squared Euclidean distance and the Ward method.

To describe the data in more detail, hierarchical clustering was performed, which was visualized using a heatmap in connection with dendrograms for relationships between species and substance classes based on the mean values for each technical triplicate (Figure 3).

Hierarchical clustering divides the dataset into two large groups and, within these, into five further clusters. One of the larger groups is represented by a cluster comprising *S. viminalis*, *S. caesia*, *S. hastata*, *S. lapponum*, *S. × sepulcralis*, and *S. daphnoides. S. bicolor* acts as the midpoint between the two larger groups, forming the second cluster. The other large group is constituted by the remaining three clusters. One of these consists of *S. cinerea*, *S. aurita,* and *S. fragilis*, whilst the other two clusters each consist of a single species, *S. purpurea* and *S. caprea*.

*S. viminalis*, *S. caesia*, *S. hastata*, *S. lapponum*, *S. × sepulcralis*, and *S. daphnoides* were characterized mainly by higher and more variable contents of SADs and CADs. *S. viminalis*, *S. caesia*, and *S. hastata* are in turn characterized primarily by high levels of CADs and flavanones. In contrast, *S. lapponum*, *S. × sepulcralis,* and *S. daphnoides* tend to exhibit higher levels of flavan-3-ols in combination with SADs. *S. bicolor*, forming the second cluster, was characterized by the highest amounts of flavones and flavanonols, moderate SAD values and surprisingly low concentrations of flavan-3-ols. The third cluster, comprising the species *S. cinerea*, *S. aurita*, and *S. fragilis*, was characterized by low levels of flavonol, CAG, and flavanonol, and, in particular, high levels of SAD. The samples of *S. caprea* differed from the others by comparatively high flavan-3-ol contents, whilst the other classes of compounds examined were present only in small quantities. *S. purpurea*, on the other hand, was characterized by high SAD and flavanone contents, as well as low flavan-3-ol contents.

Additionally, the dendrogram for substance classes also indicated relationships through five clusters. CADs and flavones often occur in higher quantities in combination, but mostly there were higher CAD than flavone levels, except for *S. bicolor*, or equal levels. It is noticeable that the substance classes of flavanonols, CAGs, and flavonols were grouped similarly as they play a minor role. In general, they were present only in small quantities in all 12 species studied, compared with the other compound classes (except for *S. bicolor*). The other three clusters, each consisting of just one substance class, are flavanone, flavan-3-ol, and SAD. Flavanones represented their own cluster due to their dominating part in a few species (*S. caesia*, *S. hastata*, *S. daphnoides*, *S. purpurea*). Flavan-3-ols and SADs outlined both relevant substance classes. The quantities of both differ considerably among the species and thus make a significant contribution to the diversity of the investigated species.

### 2.6. Correlations

To investigate relationships between the phenolic compound classes during the growth period, Pearson correlation coefficients (r) have been calculated (Table 6). Correlation coefficients were interpreted as weak (|r| < 0.3), moderate (0.3–0.5), or strong (> 0.5) and only statistically significant results are reported below.

Overall, both positive and negative correlations were observed among the different compound classes, with varying strengths. The concentrations of SAD showed a weak negative correlation with CAG (r = −0.109, *p* < 0.05) and a moderate negative correlation with flavan-3-ol (r = −0.361, *p* < 0.001), whereas the SAD and flavanone contents exhibited a moderate positive correlation (r = 0.366, *p* < 0.001). For CAD, there was a moderate negative correlation with flavan-3-ol (r = −0.374, *p* < 0.001) and a weak negative correlation with flavanone (r = −0.173, *p* < 0.001). In contrast, a weak positive correlation between CAD and the contents of flavone (r = 0.176, *p* < 0.001) and flavonol (r = 0.119, *p* < 0.05) were found. Regarding the concentration of CAG, weak negative correlations with the flavan-3-ol (r = −0.107, *p* < 0.05) and flavanone (r = −0.161, *p* < 0.001) contents were detected, while flavanonol (r = 0.223, *p* < 0.001) and flavonol (r = 0.352, *p* < 0.001) were positively correlated with CAG. Consistently weak to moderate negative correlations were observed for flavan-3-ol with every other phenolic compound class, including SAD (r = −0.361, *p* < 0.001), CAD (r = −0.374, *p* < 0.001), CAG (r = −0.107, *p* < 0.05), flavanones (r = −0.183, *p* < 0.001), flavanonols (r = −0.143, *p* < 0.01), flavones (r = −0.173, *p* < 0.001), and flavonols (r = −0.129, *p* < 0.01). The compound class of flavanones exhibited a weak negative correlation with flavonols (r = −0.118, *p* < 0.05). Meanwhile, correlation analysis concerning the concentration of flavanonol revealed a strong positive correlation with the flavonol content (r = 0.566, *p* < 0.001). Lastly, flavones and flavonols were moderately positively correlated (r = 0.214, *p* < 0.001). For the remaining correlations, no statistically significant correlations were discovered.

## 3. Discussion

### 3.1. Interspecific Variability

Twelve willow species were analyzed to examine interspecific content differences within different phenolic compound classes. The content of phenolic secondary metabolites in *Salix* is not well documented in the literature, particularly for the leaves and species that are typically less studied. This research is mostly focused on the SAD and phenolic glucoside content and more comprehensive studies have primarily examined the bark or shoots [32,33,34,35,36,37,38,39,40,41]. However, research on interspecific variability consistently reveals distinct variations in the spectrum of phenolic compounds among various species [36,37,38,39,40,43,46,67,68].

Gligorić et al. [43,47] have described the quantification of various caffeic acid derivatives for *S. fragilis* and *S. purpurea*. Compared with *S. purpurea*, it is assumed that *S. fragilis* is a species that contains a higher content of caffeic acid derivatives [43]. Another study showed that the chlorogenic acid content among the species examined was highest in *S. pentandra* (which was not examined here) and *S. fragilis*. Whereas *S. cinerea*, *S. phylicifolia*, *S. caprea*, and *S. viminialis* displayed very low levels, and *S. purpurea* and *S. lapponum* reported no chlorogenic acid, which is consistent with these results (Table 1, Figure S1A) [45].

In this study, the group of CAGs included both, coumaryl alcohol glycosides (e.g., vimalin) and acetophenones (e.g., picein). The quantification of this summarized substance class could not be found in any other publication concerning willow leaves. Comparison with previous findings is challenging because either individual coumaryl alcohol glycosides and acetophenones [46] were quantified or, for example, the substance class of phenylpropane (acids) [40], which also includes caffeic acid derivatives. Julkunen-Tiitto [46] showed that *S. cinerea* and *S. phylicifolia* contain higher levels of vimalin and triandrin, respectively, and that *S. aurita* contains relatively larger amounts of picein. Both coumaryl alcohol derivatives and acetophenones were detected in *S. viminalis*. The present results are largely consistent with the findings of Julkunen-Tiitto, except for *S. cinerea* (Table 1, Figure S1B). Only small amounts of CAG were detectable in this species. According to Wiesneth [40], *S. fragilis* displayed the highest phenylpropane acid content, while *S. daphnoides* had the lowest. The findings of this study indicate that *S. purpurea* has the lowest CAG concentration and *S. bicolor* the highest (Table 1, Figure S1B). The reason for this discrepancy is probably that *S. fragilis* revealed the highest concentrations of CAD in this analysis, the components of which are also a part of the quantified substance class phenylpropane acids [40], while *S. bicolor* was not examined at all.

The present findings regarding the flavan-3-ol content align closely with previous research (Table 1, Figure S1C). *S. caprea* and *S. cinerea* are described as species that contain high amounts of flavan-3-ols, *S. lapponum*, *S. viminalis*, and *S. aurita* with moderate flavan-3-ol levels, whereas *S. fragilis* contains low levels [43,46]. Gligorić et al. [43] showed that the flavan-3-ol content of *S. purpurea* is about twice as high as in *S. fragilis*, which can be confirmed here (Table 1, Figure S1C). In another publication by Gligorić et al. [47], the opposite was shown, as also described by Wiesneth [40], which is probably due to different extraction methods. However, as the method of Wiesneth [40] was applied in this study, the comparison cannot be ascribed to differences in extraction or quantification. The variations are possibly more likely to be caused by seasonal and climatic factors [34,47]. Substantial variation in the levels of flavan-3-ol compounds has been reported across different willow culture forms and locations [69], as also demonstrated in this study (Table 1 and Table 4). Flavan-3-ols also count as potent antioxidants [70]. It has been noted that catechin concentrations vary in response to different stressors, like water scarcity [71], and that they shield plants from pathogens like bacteria, fungi, insects, and herbivores [17,72,73].

Very few studies have quantified the flavonoid content and divided it into the various flavonoid subclasses, especially not for less studied species [74]. Either individual flavonoids or the total flavonoid content were typically measured [40,43,47]. In general, the preferred deposition site for flavonoids are the leaves [47]. Nevertheless, while flavonols preferentially accumulate in leaves, flavanones tend to accumulate in the shoots [40]. The present data confirm that *S. purpurea* is an exception to this (Table 2 and Table 3, Figure S1D). Each of the other species only showed relatively small amounts of flavanones in the leaves, suggesting the accumulation in the shoots, whereas *S. purpurea* contained much more. Studies by Gligorić et al. [43,47] have also shown that the concentration of naringenin, which is a flavanone, was highest in *S. purpurea*. The group of flavonols and flavones compiled as flavonoids has been quantified by Wiesneth [40]. Thereby, *S. daphnoides* was found to have the highest flavonoid content. The present study was unable to prove this, as the highest flavonol and flavone contents in these data were found in *S. bicolor*, which was not analyzed by Wiesneth (Table 3, Figure S1F,G). Nevertheless, Vanhakylä and Salminen also showed that *S. bicolor* has a high flavonol content [75]. The flavonol content of *S. daphnoides* was rather in the lower range in this analysis, whereas the flavone content was rather in the upper range. Overall, after *S. bicolor*, the highest flavonoid content (the sum of the flavonol and flavone contents) was detected in *S. purpurea*, followed by *S. fragilis*, which has also been demonstrated by Gligorić [47]. Additionally, rutin and quercetin, which are classified as flavonols, are found in high concentrations in *S. purpurea*, at least in comparison to *S. fragilis* [43,47]. The present results are similar to this, as substantially higher amounts of flavonols were detected in *S. purpurea* compared with *S. fragilis* (Table 3, Figure S1G). As the species examined in this study are less investigated and have a very low flavanonol content, no literature on the subject could be found.

The genus *Salix* has a wide spectrum of flavonoids, which differ depending on the species (Table 1, Table 2, Table 3, Table 4 and Table 5), especially in the leaves [5]. They, according to quercetin, contribute to many functions, like acting as an allelochemical and antifungal agent, as a pollinator-attracting pigmentation, and as a signaling molecule in plant–insect interactions [73]. Several willow genotypes have been found to exhibit notable differences in quercetin levels [76].

Comparability to previous studies is generally challenging, not only due to different methods and obtained results, but also due to seasonal fluctuations. If samples are harvested only at a certain time point within the growing season, seasonal fluctuations are not fully captured, which complicates species characterization given the high seasonal and interannual variability reported in the literature [20,32,36,40,44]. The calculation of mean values has resulted in relatively high standard deviations for several species (*S. caprea*, *S. cinerea*, *S. daphnoides*, *S. fragilis*, and *S. purpurea)*, as multiple individuals per species were analyzed (Table 1, Table 2 and Table 3). This variability can be attributed to differences in the phenolic compound contents depending on the harvest time and due to impurely assigned peak areas. Additionally, unequal sample sizes among species may lead to certain species exerting an imbalanced impact on overall patterns. To address this issue, species were treated as fixed factors in the statistical analysis, whilst individuals were modeled as random factors nested within species. This approach minimizes the bias introduced by more frequently sampled species and ensures that interspecific differences are appropriately represented. By quantifying compound classes, the current study attempted to avoid the variability of individual substances [32,43,47].

The fact that all studied individuals originate from a botanical garden represents a controlled yet limiting framework. On the one hand, these conditions reduce environmental variability (e.g., soil, water availability, competition), thereby revealing species-specific differences in phenolic profiles more clearly. This is a methodological advantage for comparative phytochemical screening, as confounding site factors are minimized. On the other hand, the transferability of the results to natural populations is limited. Under natural conditions, *Salix* species are exposed to considerable ecological and genetic variability, which is known to strongly influence secondary metabolites. Factors such as habitat conditions (e.g., moisture, nutrient availability), biotic stress (herbivory, pathogens), climatic influences, and intraspecific genetic diversity can significantly influence the accumulation of phenolic compounds. Based on this, it can be assumed that the phenolic profiles observed in this study are likely to be representative of the investigated species. Furthermore, botanical gardens represent a limited genetic sample, which may lead to an underestimation of intraspecific variation. Despite these limitations, the results remain meaningful, particularly regarding the identification of fundamental species-specific trends and potential chemotaxonomic patterns. However, wide-ranging conclusions concerning the entire genus *Salix* should be interpreted with caution.

In summary, these results, together with the previous analysis [38], show that the levels of the investigated phenolic compound classes vary in a highly significantly way between species (Table 1, Table 2, Table 3, Table 4 and Table 5).

### 3.2. Interannual Variability

To investigate the effect of harvest year on the phenolic compound contents, samples were obtained during the growth season in two separate years.

In the limited available research, different results concerning interannual variability are documented. On the one hand, no significant differences were found between subsequent harvest years related to the phenolic content in the leaves of *S. myrsinifolia* [48]. On the other hand, a decreasing SAD content was detected in the leaves of older plants [49]. However, the authors did not examine the same individuals in successive growth periods, but rather individuals of different ages. In contrast, increased concentrations of phenolic compounds in leaves in the following year are attributed to an increased biomass and the associated increase in synthesizing tissue [50]. Other studies relate mainly to the bark and are therefore not comparable [51,52,53]. This study showed partially contradictory observations. Concerning the CAD content, it was higher in the subsequent year and for flavan-3-ol level it was the opposite. For the other substance classes, there was no significant trend (Table 4 and Table 5).

It is challenging to distinguish the effects of harvest years from climatic variations, and the short investigation period (two years) restricts interpretability. To increase statistical power, the harvest year was considered as a fixed factor whilst individuals were modeled as random factors nested within species in the linear mixed effects model, which made it possible to evaluate annual trends, whilst simultaneously considering the unbalanced sampling, even though species-specific patterns may be partly obscured.

Together with the previous analysis of the SAD content [38], these results confirm the assumption that the variability in phenolic contents is not only very high within a growing season, but also as the years progress with regard to the CAD and flavan-3-ol contents.

### 3.3. Interseasonal Variability

Seasonal investigations on the content of different phenolic compound classes were carried out during the growing seasons of 2018 and 2019 to study interseasonal variability.

The caffeic acid derivatives and coumaryl alcohol glycosides (including acetophenones) are discussed together below due to a lack of comparable literature. According to the research, phenylpropane acid levels peak in May and display a minimum in September [40]. This pattern was also detectable for the substance class of CADs (Table 4). Again, *S. purpurea* is an exception, with a maximum found in August. Another exception was *S. daphnoides* with a maximum in August/September [40], which could not be confirmed.

The pattern of the CAG content was contrary to the CAD levels in the present study (Table 4) and also to the literature regarding phenylpropane acids. In this case, the minimum content was mostly found in May, and the maximum was consistently reached at the end of the growing season.

Regarding the flavan-3-ol contents, the trend is consistent with the literature concerning the fact that some species tend to show a maximum towards the beginning of the growth period, others towards the end [39,40] (Table 4). The situation is different for the minima. These are mainly found in May or July. However, there are a few similarities in the species-specific maxima and minima. As the same extraction and quantification method was used here as in the literature [39,40], these differences can be attributed to climatic and seasonal differences. However, using statistical analysis, it was possible to demonstrate that the content is significantly higher at the end of the growing season (Table 4).

Concerning the flavanone content, the present findings confirm the observations of Wiesneth [40]. The minimum is predominantly found at the beginning of the growth period and the maximum, though not as obviously, towards the end (Table 5). *S. fragilis* and *S. daphnoides* deviate from this pattern. In contrast, this study presented different results for *S. caprea* and *S. cinerea* than the literature [40] (Table 5). For *S. caprea*, a maximum was detected at the beginning of the growth period, and a minimum at the end, and vice versa for *S. cinerea*. Nevertheless, using statistical analysis, it could be demonstrated that the content had significantly increased by the end of the growth period (Table 5).

The same applies to flavonols and flavones. Summarized as flavonoids, these show a similar pattern when compared with the flavanone content [40]. However, concerning species-specific minima and maxima, there are few similarities. *S. fragilis* and *S. purpurea* display their maxima at the end of the growth period (Table 5, Figure S1G). For both classes of constituents, *S. caprea* and *S. daphnoides* exhibit maxima in August and September, which is not consistent with Wiesneth [40]. The reverse is also the case for *S. cinerea*. It has also been demonstrated that the flavonol concentration in *S. bicolor* is either fairly stable over the growth period or may slightly decline. This trend can only be partially confirmed (Figure S1G). However, this statement is also not statistically significant [75] (Table 5). Due to a lack of literature, there are no comparable statements on the flavanonol content.

Monthly samples, which were collected on one day per month, cannot accurately reflect seasonal variations because they are affected by environmental variables. Therefore, the harvest month was treated as a fixed factor in this model and individuals nested within species as random factor. This mixed effects model allowed for a reliable analysis of overall seasonal trends, taking into account species identity, individual variability and unbalanced sampling, even though species-specific temporal patterns may be partially masked.

In summary, it can be concluded that the content of phenolic secondary metabolites in the leaves of the analyzed willow species is significantly higher towards the end of the growing season, for most of the phenolic compound classes examined, similar to the prior study on SAD content [38] (Table 4 and Table 5). Late-season harvesting may be preferable for maximizing phenol-rich content, although species-specific variation must be considered.

Once again, it becomes clear how high the seasonal variability is within the genus *Salix*. The latter is further underlined by the fact that even results from the same individuals collected from the same location in different growing seasons and examined using the same methods do not fit together [40].

### 3.4. Intersexual Variability

The species *S. caprea*, *S. cinerea*, *S. daphnoides*, *S. fragilis*, and *S. purpurea* were analyzed regarding the intersexual variability within each phenolic compound class.

There are different observations about whether sex has an influence on the phenolic compound spectrum or not. According to Nissinen et al., female leaves had higher contents of flavonoids, whereas male leaves contained more SADs [54,55]. Moreover, the sex ratio is biased toward females in the genus *Salix* [77,78]. Males tend to be more vulnerable to herbivores [79,80] and have lower survivability [55,81]. In contrast, females tend to be less sensitive to insects [79,82], have higher levels of defensive compounds, like salicylic alcohol derivatives, flavonoids, and tannins [16,83,84], and have higher biomass [85,86]. Although Yang [55] acknowledged this pattern, he concluded that a higher phenolic content in females is not primarily for defense. Rather, it is connected to the higher nitrogen content of female leaves, which may shield insects. Nevertheless, other research has shown that there are no significant variations in the phenolic content spectrum between the sexes [38,56,57,58,59], which can be confirmed (Table 4 and Table 5).

The unequal sex distribution in the samples of this study across some species restricts the conclusions that can be made about sex-specific effects, though linear mixed models with individuals nested within species were used to account for the unbalanced number of individuals. This unbalanced sampling diminishes statistical power to identify minor to moderate sex-specific variations. It additionally elevates the chance that species-specific or site-specific effects will skew sex-associated trends. Consequently, the lack of significant intersexual differences should be regarded cautiously. Evidence requires an increased number of samples and a more equal distribution of sexes per species.

### 3.5. Phytochemical Classification

Although several clustering methods show a different number of clusters, many similarities can be recognized (Figure 1, Figure 2 and Figure 3). Based on all of these results, it is clear that *S. bicolor* exhibited a completely different composition of the phenolic component spectrum. For *S. purpurea* and *S. caprea*, this was comparable. Furthermore, the other species seem to be more similar to each other. Nevertheless, subordinate clusters still emerge here, one consisting of *S. aurita*, *S. cinerea*, and *S. fragilis*, one constituted of *S. × sepulcralis* and *S. daphnoides*, and one consisting of *S. viminalis*, *S. caesia*, *S. hastata*, and *S. lapponum* (Figure 3).

Compared with the literature, there are many different classifications [1,46,47,57,60,62,87,88,89,90]. A common morphological classification by Skvortsov divided the genus *Salix* into 3 subgenera and 26 sections [60]. Two of the 12 species examined in this study are classified in the subgenus *Salix*, namely *S. fragilis* and *S. × sepulcralis*. All of the other species belong to the subgenus *Vetrix*. Based on the clustering methods, the present data do not confirm this in many points (Figure 1, Figure 2 and Figure 3). *S. fragilis* and *S. × sepulcralis* are not consequently grouped in the same cluster. In addition, *S. purpurea* and *S. caesia* are commonly not clustered together, despite Skvortsov asserting that they are in the same section within the subgenus *Vetrix*. However *S. cinerea* and *S. aurita* belong to the same cluster [60].

Recent phylogenetic studies indicate that there are just two subgenera [1,62,89,90,91,92,93,94]. These investigations cluster the genus *Salix* into the subgenera *Salix* and *Vetrix,* whereas subgenus *Vetrix* includes subgenus *Chamaetia*. This is an observation that could also be applied to the present results. The PCA, as well as the hierarchical clustering, display two large clusters. One of these consists either of *S. bicolor* or *S. purpurea* and the other cluster of all of the other species. However, the phylogenetic classification clearly shows that the subgenus *Salix* includes *S. fragilis* and species such as *S. bicolor* and *S. purpurea*, which are clearly distinguished from the other species in this study, are located in the subgenus *Vetrix*. However, a closer look at the relationships within the subgenus *Vetrix* shows that *S. cinerea* and *S. aurita* are closely related, which is also reflected in the presented results (Figure 3) [89,90]. There are also phylogenetic studies in which *S. caprea* and *S. aurita* are closely related [1,60]. Furthermore, it has been shown that *S. lapponum* and *S. viminalis* are closely related [89,90]. Ogutcen et al. [89] have also pointed out that *S. caesia* and *S. purpurea* are closely related, according to Skvortsov [60], though this is a pattern that could not be confirmed either (Figure 3).

Concerning other phytochemical classifications, there are similarities, as well as differences. All species evaluated in this study were included in the investigations of Julkunen-Tiitto, apart from *S. caesia* [46]. *S. purpurea* was clustered in one cluster, *S. daphnoides* and *S. hastata* together in another cluster, and the other analyzed species in a third cluster. *S. purpurea* is thus also distinguished from the other species here. It is striking that *S. fragilis* is clustered here, as in the present studies, together with most other species, and is not strongly differentiated. Furthermore, according to Julkunen-Tiitto, *S. daphnoides* and *S. hastata* are classified in one cluster, which can be confirmed (Figure 3). The remaining species form the third cluster in the study by Julkunen-Tiitto [46], except for *S. cinerea*. Gligorić et al. also grouped the investigated species into three groups, whereby one cluster consisted of *S. purpurea*, similar to Figure 3, the second cluster included *S. fragilis*, and the third one contained all of the other species (including none of the species analyzed in this study) [47]. In contrast, Nyman and Julkunen-Tiitto clustered *S. lapponum* and *S. bicolor* in the same cluster, which cannot be directly confirmed [20].

As both methods used in this study are based on essentially different mathematical principles, differences between the clusters generated are not surprising. This is because the purpose of PCA is to explain the maximum variance by reducing the dimensionality (in this analysis, only the first two principal components were plotted). Hierarchical clustering, on the other hand, considers differences that may not be apparent in the principal components, as it uses the entire multidimensional distance matrix. Consequently, species may be grouped independently in the dendrogram but be close to each other in the PCA plot. These differences show that both methods highlight complementary features of interspecific similarity and that the detectable pattern of the data depends on the methodology used.

In the past, chemotaxonomic analysis often yielded different results than phylogenetic investigations [20]. Even different studies on a chemical basis led to different results, partly due to the selected analyzed compounds, analytical and statistical methods, as well as the studied species. These suggest that phytochemical classification alone is not very useful [20,95]. In addition, the genus *Salix* is extremely difficult to classify morphologically [60,61] or phylogenetically [91,92]. Nevertheless, phylogenetic analysis has demonstrated that the examination of genes, which mainly correlate with the morphology, can yield more meaningful relationships [1,62]. A phytochemical classification on a quantitative basis is insufficient. Additional qualification and/or the identification of marker substances could be helpful, or a combined phylogenetic and chemotaxonomic classification would provide more helpful information, as even small genetic changes within a species can lead to major changes in phytochemical properties [96,97,98,99].

### 3.6. Correlation

The correlation analysis aimed to explore potential relationships between phenolic compound classes in the context of their biosynthetic relations during the growth period. As the available number of observations per species was limited, Pearson correlation coefficients were calculated across the entire dataset, including all species, individuals, sampling months (May–September), and both years (2018–2019) (Table 6). This allowed for the identification of general patterns across the genus *Salix*, while acknowledging that species-specific correlations could not be robustly assessed due to insufficient statistical power at that level.

For this purpose, several correlations are biosynthetically plausible. The other correlations are not of biosynthetic interest and will not be discussed here.

CADs represent important intermediates within the phenylpropanoid pathway and are substrates of the chalcone synthase. Via this enzyme, CADs are linked with a phloroglucinol to form a chalcone and then converted into flavanones via the chalcone isomerase [100]. Therefore, species with high flavanone concentrations and low CAD content may exhibit high enzyme activity, i.e., as for *S. purpurea* [40]. This hypothesis is supported by statistical analysis, which revealed a significant negative correlation between the CAD and flavanone contents (Table 6).

Flavone synthase can then convert these flavanones into flavones, and flavanone-3-hydroxylase further converts them into flavanonols. These can then be transformed into flavonols or flavan-3-ols, for example, by various enzymes [100]. A low CAD content could therefore also be associated with high concentrations of these flavonoid classes because of the increased activity of the enzymes involved. These assumptions are supported by the significantly negative correlation coefficients presented in Table 6. The CAD content correlates negatively not only with the flavanone content, but also with the flavan-3-ol content. Indicating that, the less CAD is contained in a species, the more flavanones and flavan-3-ols it contains (or vice versa), which could be related to a modulated activity of the enzymes involved.

Additionally, there is a negative correlation between the flavanone content and the flavonol and flavan-3-ol contents (Table 6). Given that flavanonols form the next step in the biosynthesis, it is remarkable that there is no detectable significant negative correlation between these two. However, this is followed by the conversion into flavonols or flavan-3-ols [100]. Indicating that, the fewer flavanones a species contains, the more flavan-3-ols and flavonols it contains (or vice versa). However, this assumption tends more towards increased activity of leucoanthocyanidin reductase, which lastly converts flavandiols into flavan-3-ols [100]. This assumption results from the higher contents of flavanones and flavan-3-ols in the various *Salix* species compared with the flavonol concentration (Figure 3), as well as on the oppositely directed vectors (PCA loading plot) of flavan-3-ols relative to the other phenolic classes, particularly flavonols, as shown in Figure 2. An exception to this is *S. bicolor*. In this case, it could be assumed that the activity of flavanone-3-hydroxylase and flavone synthase is increased, while dihydroflavonol-4-reductase and flavonol synthase are inhibited.

Further along in the biosynthesis, there is a negative correlation between the flavanonol content and the flavonol and flavan-3-ol content (Table 6). In general, this suggests that the flavonol synthase is more likely to be less active than the dihydroflavanol reductase, because none of the examined species showed a notable flavonol content, whereas the opposite was the case for the flavan-3-ol concentration (Figure 2 and Figure 3).

The flavan-3-ol content correlated negatively with all flavonoid classes (Table 6), which is also reflected in the PCA (Figure 2) by their contrasting loading direction compared with the other compound classes. As the biosynthesis of flavonoids nearly terminates there, this could indicate that both the availability of the precursor flavonoids in biosynthesis and the activity of the involved enzymes are necessary for formation [100]. This may suggest that the accumulation of flavan-3-ols represents a distinct metabolic endpoint under certain physiological or environmental conditions.

From a biosynthetic perspective, the relationship between CAD and SAD content is also particularly interesting because CAD is a precursor to SAD [101]. Phenylpropanes are used to form the salicyl and HCH components of the majority of SADs. Statistical analysis, however, revealed no correlation between these phenolic compound classes (Table 6), suggesting that this process is more complicated and is influenced by several factors and therefore not directly captured by simple correlation analysis.

In general, the formation of flavonoids and phenolic acids is a sign that the shikimate pathway is activated. This could be caused, for instance, by increased metal concentrations in the soil, as it induces various enzymes such as peroxidase and shikimate dehydrogenase [102,103,104,105]. The presence or absence of specific insects, to which some species or individuals react with defense systems, may also be indicated by the increased activity of specific enzymes [14,16,82,106,107,108].

The correlations, presented in Table 6, between individual substance classes are consistent with known biosynthetic relationships and suggest possible precursor–product relationships or common regulatory mechanisms. However, as correlations do not allow causal conclusions to be drawn, these are simply hypotheses that require further investigation. To prove actual metabolic conversions, further investigations, like analyses of enzyme activity or isotope-labelled experiments, would be necessary.

## 4. Materials and Methods

### 4.1. Plant Material

The sample collection and selection of species and individuals were the same as in the previous study [38]. Plants were located at the Ecological-Botanical Gardens of the University of Bayreuth (ÖBG Bayreuth), Germany. The screening included the following 12 Central European *Salix* species from various related groups within the genus: *Salix aurita* L., *S. bicolor* Ehrh. ex Willd., *S. caesia* Vill., *S. caprea* L., *S. cinerea* L., *S. daphnoides* Vill., *S. fragilis* L., *S. hastata* L., *S. lapponum* L., *S. purpurea* L., *S. viminalis* L., and *S. × sepulcralis* Simonk., each grown under comparable environmental conditions. In total, 42 individuals (15–25 years old) were included.

Among these, the number of individuals per species varied. *S. caprea*, *S. cinerea*, *S. fragilis*, and *S. purpurea* were represented by more than eight individuals in the Ecological Botanical Garden. Thus, eight individuals per species were selected for these investigations. For all other species, all available individuals were sampled, which corresponded to one individual per species, apart from *S. daphnoides*, of which three individuals were present and investigated.

For species represented by several individuals, the aim was to achieve a balanced sex ratio. This was the case for all species except *S. purpurea* (3 males, 5 females) and *S. daphnoides* (2 male, 1 females), as it was logistically impossible to achieve a balanced ratio for the latter.

Species identification was verified by Gregor Aas [4,109]. Voucher specimens of all investigated individuals were deposited in the herbarium ID UBT/Ecological-Botanical Gardens of the University of Bayreuth (ÖBG Bayreuth), Germany. The corresponding voucher numbers are provided in Table S1, as well as the number of individuals per species, sex, intern identification number, GPS coordinates, and common synonyms.

Soils were classified as clay and sandy loam with a shallow humus-rich top layer (mull-like Moder), with pH (H_2_O) ranging from 4.4 to 5.3. Climatic data during the investigation period (Figures S2–S5) and photographs of all species are available in the Supplementary Data (Figures S6–S17).

### 4.2. Sample Preparation

Sampling of the leaves was carried out, as in the previous study [38], between May and September 2018 and 2019 on the following dates: 7 May 2018, 11 June 2018, 2 July 2018, 30 July 2018, and 3 September 2018, as well as 13 May 2019, 3 June 2019, 1 July 2019, 5 August 2019, and 10 September 2019, except for *S. hastata*. This individual died after the sampling in May 2019. The plant material was then cut and vacuum-dried over silica gel (Carl Roth GmbH + Co. KG, Karlsruhe, Germany). Afterward, it was ground using a swing mill MM400 (Retsch GmbH, Haan, Germany) and stored at −10°C until further use. Each sample consisted of leaves collected from a single individual at a given time point (e.g., May 2018), representing one biological replicate. Three technical replicates were analyzed per sample.

### 4.3. Extract Preparation for UPLC^®^ Analysis

For the extraction of the phenolic compounds, a method described by Wiesneth was used [40]. In summary, 1.00 mL MeOH with Citropten (0.2 mg/mL, internal standard) was added to 50 mg of the dried plant material. Samples were then extracted in a supersonic bath (VWR International GmbH, Ismaning, Germany) at room temperature for 30 min. Afterward, they were centrifuged (Sigma GmbH, Osterode am Harz, Germany) at 14,000 rpm for 3 min. Before analysis, samples were passed through a 0.2 µm Perfect Flow^®^ RC Filter (WICOM Germany GmbH, Heppenheim, Germany).

### 4.4. UPLC^®^ Analysis

The separation process was conducted using a Waters ACQUITY UPLC^®^ (Waters GmbH, Eschborn, Germany), which included an ACQUITY H-Class QSM, FTN, and PDA detector. Reversed-phase separation was carried out on a Luna^®^ omega C18 column (1.6 µm, 100 Å, 100 × 2.1 mm, Phenomenex, Aschaffenburg, Germany) with a column temperature of 50°C, including an active preheater. The applied gradient, with a flow rate of 0.5 mL/min, where eluent A consisted of H_2_O with 1% formic acid (Carl Roth GmbH + Co. KG, Karlsruhe, Germany) and eluent B consisted of ACN (Merck KGaA, Darmstadt, Germany) with 1% formic acid, was as follows: 0.0–0.5 min 5% B isocratic; 0.5–9.0 min 5–30% B; 9.0–10.5 min 30–50% B; 10.5–11.5 min 50–100% B; 11.5–14.0 min 100% B isocratic; 14.0–15.0 min 100–5% B; and 15.0–17.0 min 5% B isocratic [40]. The injection volume was 1.0 µL and UV spectra were recorded between 200 and 400 nm, with quantification at 279 nm.

**Figure 4:**
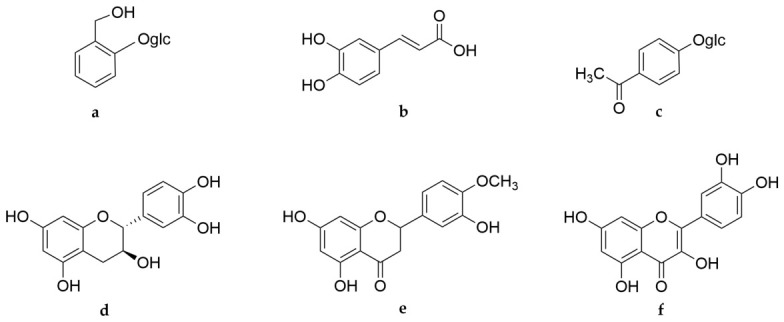
Structures of reference compounds used in 4.4. (**a**) Salicin, (**b**) caffeic acid, (**c**) picein, (**d**) catechin, (**e**) hesperetin, and (**f**) quercetin; glc = glucose.

To determine the content of the phenolic components studied, a calibration curve was created for citropten (SIGMA-ALDRICH GmbH, Steinheim, Germany) (0.2–2 mmol/L, internal standard). Three stock solutions were prepared for the calibration, each with two dilution series in MeOH (Merck KGaA, Darmstadt, Germany). The following standard compounds were used as references (Figure 4): salicin (a) (Carl Roth GmbH + Co. KG, Karlsruhe, Germany) for substance class SAD [38], caffeic acid (b) (Carl Roth GmbH + Co. KG, Karlsruhe, Germany) for substance class CAD, picein (c) (PhytoLab GmbH & Co. KG, Vestenbergsgreuth, Germany) for substance class CAG, catechin (d) (SIGMA-ALDRICH GmbH, Steinheim, Germany) for substance class flavan-3-ol, hesperetin (e) (ThermoFischer (Kandel) GmbH, Karlsruhe, Germany) for substance classes flavanone and flavanonol, and quercetin (f) (SIGMA-ALDRICH GmbH, Steinheim, Germany) for substance classes flavone and flavonol. These were dissolved in mixture in MeOH with citropten (0.2 mg/mL) and three stock solutions were prepared for the calibration, each with two dilution series. Correction factors were calculated to allow for quantification according to Wiesneth [40].

Peak identification was carried out using a spectral database via UV maxima for automatic substance assignment according to the spectral contrast theory (SCT) by comparing the recorded UV spectra of individual peaks with reference spectra of the database [37]. The wavelength range of 230–400 nm was selected for spectral matching. This restriction was imposed to exclude highly noisy spectral regions and regions with higher wavelengths (>400 nm), in which most analytes exhibited only minimal absorption. Including these regions of low absorption would artificially reduce the match angle calculated by the SCT, thereby decreasing spectral resolution and compromising the reliability of automated peak classification. The chromatograms were automatically integrated using the traditional integration function in Empower3 at a detection wavelength of 279 nm. This wavelength provided consistent peak integration across the different compound classes, which simplified the automated processing and comparative analysis of large datasets, even though they differ in their individual absorption maxima.

Each sample was analyzed in triplicate and the analyte content was calculated in mg/g dry weight. The standard deviation of mean values for species represented by multiple individuals *(S. caprea*, *S. cinerea*, *S. daphnoides*, *S. fragilis*, and *S. purpurea*) demonstrates intraspecific variability, while the standard deviation of mean values from technical triplicates reflects measurement precision. 

### 4.5. Statistical Analysis

The statistical analysis of this study, concerning the variability, was conducted in the same manner as in the previously published work [38] and was performed using R Version 4.2.3 [110]. First, the mean value of each triplicate was calculated. Then, for the linear mixed models, the concentrations of each substance class were log-transformed.

Data were analyzed using linear mixed models, with individuals nested within species as random factor, unless otherwise specified. All linear mixed models were analyzed using the lmer() function in the lme4 [111] and lmerTest [112] package.

To evaluate the overall influence of year, month, and sex, on each substance class these variables were treated as fixed factors following a type II ANOVA. The ANOVAs were done using the Anova() function in the car package [113].

For comparisons among species and to investigate the overall influence of species, the variables species, month, and year were used as fixed factors, with individual as random factor, to not disregard the influence of month and year. Moreover, exclusion of the intercept ensured that individual estimates were obtained for each species. A type II ANOVA followed by Tukey’s honest significant difference (HSD) test (*p* < 0.05) was subsequently carried out. For the Tukey HSD test, the functions glht() and cld() in the packages multcomp [114] and multcompView [115] were used.

Furthermore, a principal component analysis (PCA) was performed to investigate similarities and differences between the analyzed species based on the content of SAD, CAD, CAG, flavan-3-ol, flavanone, flavanonol, flavone, and flavonol using the prcomp() and biplot() function, as well as the autoplot() function in the ggfortify package [116,117].

Using the ward method and squared Euclidean distances, hierarchical clustering analysis was also used to compare the tested samples with the function heatmap().

Pearson correlation coefficients were used to investigate the relationships between the different phenolic compound classes with the cor() function and were calculated across the entire dataset, including all species, individuals, sampling months (May–September), and years (2018–2019).

The package dplyr was used to prepare the data for analysis [118] and ggplot2 to create the graphics [119]. *p* values < 0.05 were regarded as statistically significant, *p* < 0.01 as very significant, and *p* < 0.001 as highly significant.

## 5. Conclusions

In summary, the present study revealed clear species-specific differences in the phenolic compound profiles of the *Salix* species examined. *S. bicolor* exhibited particularly high levels of CADs, CAGs, flavones, flavanonols and flavonols, whilst *S. caprea* was characterized by exceptionally high flavan-3-ol levels and *S. purpurea* by elevated flavanone and SAD concentrations. This was also demonstrated by the results of principal component analysis and hierarchical clustering, with *S. bicolor*, *S. purpurea,* and *S. caprea* displaying distinct metabolite profiles.

In this regard, correlation analysis identified biosynthetically plausible relationships between several phenolic compound classes. Significant negative correlations between CADs and flavanones as well as flavan-3-ols could indicate differences in enzyme activity within the biosynthetic pathways of phenylpropanoids and flavonoids. Similarly, the differing patterns of flavan-3-ols, compared with other flavonoid classes, indicate that the formation of flavan-3-ols could serve as a metabolic endpoint under particular physiological or environmental conditions. These conclusions remain hypothetical and require confirmation by targeted biochemical and enzymatic studies.

In addition to the interspecific variations, seasonal effects significantly influenced the accumulation of phenolic compounds. The harvest time had a considerable effect on the content of almost all analyzed compound classes, reaching maximum concentrations towards the end of the growing season. Interannual differences were also detected, especially for CADs and flavan-3-ols, whilst the influence of sex was generally weak and statistically not significant.

With a wide range of species distribution and significant seasonal and species-specific variation, the diverse willow species provide a rich source of phenolic compounds. For sectors ranging from biorefineries and environmental applications to pharmaceuticals and nutraceuticals, this offers a variety of opportunities. Instead of concentrating only on salicylic alcohol derivatives, it is essential to examine the entire range of phenolic compounds found in various willow species to improve the development of willow-based phytopharmaceuticals.

Chemotaxonomical screenings yield valuable information about the species-specific secondary metabolite profiles of willows. Research on species that are not widely investigated for phytopharmaceutical aspects, in particular, helps to expand the knowledge of the phenolic compound profiles. Selecting species and additional plant parts, such as leaves, strategically can enhance sustainability, promote the circular bioeconomy, and maximize resource use. Relationships within the genus *Salix* may become clearer by combining phytochemical and phylogenetic research. However, comparison of phytochemical and phylogenetic relationships should be treated with caution [20,95]. The controlled breeding of willow species with an optimized phenolic content spectrum and high-biomass represents a possibility [120,121,122]. Further research on interseasonal, interannual, and intersexual variability is necessary to determine precisely what affects the profile of phenolic compounds. The same applies to the influence of herbivores and the activation of enzymes involved in the biosynthesis of these components.

## Figures and Tables

**Table 1:** Mean values and corresponding standard deviations of CAD, CAG, and flavan-3-ol contents in mg/g DW, as well as results of linear mixed models. Comparison of the species in terms of CAD, CAG, and flavan-3-ol contents with Tukey’s HSD test. R^2^ represents the average log-transformed concentration estimates of the content in mg/g DW without reference to the other species.

	CAD				CAG				Flavan-3-ol			
	Content		Effect		Content		Effect		Content		Effect	
Species	Mean	SD	R^2^	SE	Mean	SD	R^2^	SE	Mean	SD	R^2^	SE
*S. aurita*	4.1	1.1	1.60 abc	0.26	0.4	0.6	0.25 abc	0.04	18	9	2.8 abc	0.4
*S. bicolor*	15	19	2.04 bd	0.26	0.5	0.5	0.38 b	0.04	2.4	1.5	1.1 de	0.4
*S. caesia*	4.0	1.2	1.58 abc	0.26	0.25	0.12	0.22 bd	0.04	2.1	2.0	1.1 de	0.4
*S. caprea*	2.3	1.3	1.14 c	0.09	0.04	0.08	0.039 ef	0.014	36	13	3.56 a	0.12
*S. cinerea*	3.5	1.4	1.47 bc	0.09	0.13	0.27	0.099 df	0.014	17	12	2.71 b	0.12
*S. daphnoides*	1.9	0.8	1.02 c	0.15	0.13	0.10	0.116 adf	0.023	11	6	2.46 bc	0.19
*S. fragilis*	15	6	2.73 d	0.09	0.05	0.16	0.045 ef	0.014	4	4	1.31 e	0.12
*S. hastata*	15	7	2.74 ad	0.27	0.19	0.24	0.17 cde	0.05	3.2	2.6	1.4 ce	0.4
*S. lapponum*	2.7	0.6	1.28 bc	0.26	0.4	0.4	0.32 bc	0.04	9	4	2.2 bce	0.4
*S. purpurea*	3.0	2.5	1.25 bc	0.09	0.02	0.05	0.017 e	0.014	8	6	1.94 cd	0.12
*S. viminalis*	4.4	1.4	1.65 abc	0.26	0.24	0.29	0.19 cd	0.04	8	5	2.1 bce	0.4
*S. × sepulcralis*	5.4	1.6	1.83 abc	0.26	0.06	0.08	0.05 de	0.04	17	12	2.6 abc	0.4

Different letters indicate interspecific significant differences in content (*p* < 0.05). SD—standard deviation; R^2^—regression coefficient; SE—standard error of regression coefficient.

**Table 2:** Mean values and corresponding standard deviations of flavanone and flavanonol contents in mg/g DW, as well as results of linear mixed models. Comparison of the species in terms of flavanone and flavanonol contents with Tukey’s HSD test. R^2^ represents the average log-transformed concentration estimates of the content in mg/g DW without reference to the other species.

	Flavanone				Flavanonol			
	Content		Effect		Content		Effect	
Species	Mean	SD	R^2^	SE	Mean	SD	R^2^	SE
S. *aurita*	6.2	1.1	2.0 abc	0.4	0.02	0.04	0.0 ab	0.4
*S. bicolor*	4.5	1.6	1.7 bc	0.4	13	15	2.0 c	0.4
*S. caesia*	7.4	1.1	2.1 abc	0.4	0.30	0.20	0.2 ab	0.4
*S. caprea*	5.1	2.1	1.73 c	0.14	0.03	0.05	0.03 a	0.12
*S. cinerea*	2.2	1.1	1.10 b	0.14	0.08	0.17	0.07 ab	0.12
*S. daphnoides*	11.6	2.3	2.52 ac	0.22	0.19	0.17	0.17 ab	0.18
*S. fragilis*	6.4	3.2	1.94 c	0.14	0.1	0.4	0.12 ab	0.12
*S. hastata*	7.0	2.6	2.1 abc	0.4	0.14	0.07	0.1 ab	0.4
*S. lapponum*	2.9	0.5	1.4 bc	0.4	2.9	3.5	1.0 ab	0.4
*S. purpurea*	29	15	3.22 a	0.14	1.30	2.31	0.55 b	0.12
*S. viminalis*	2.7	0.8	1.3 bc	0.4	0.07	0.13	0.1 ab	0.4
*S. × sepulcralis*	3.5	1.3	1.5 bc	0.4	0.011	0.024	0.0 ab	0.4

Different letters indicate interspecific significant differences in content (*p* < 0.05). SD—standard deviation; R^2^—regression coefficient; SE—standard error of regression coefficient.

**Table 3:** Mean values and corresponding standard deviations of flavone and flavonol contents in mg/g DW, as well as results of linear mixed models. Comparison of the species in terms of flavone and flavonol contents with Tukey’s HSD test. R^2^ represents the average log-transformed concentration estimates of the content in mg/g DW without reference to the other species.

	Flavone				Flavonol			
	Content		Effect		Content		Effect	
Species	Mean	SD	R^2^	SE	Mean	SD	R^2^	SE
*S. aurita*	2.0	0.8	1.06 ab	0.25	0.3	0.4	0.22 a	0.16
*S. bicolor*	24	31	2.17 a	0.25	5	2	1.71 b	0.16
*S. caesia*	3.1	1.3	1.38 ab	0.25	0.11	0.2	0.10 a	0.16
*S. caprea*	1.7	1.5	0.95 b	0.09	0.11	0.21	0.09 a	0.06
*S. cinerea*	3.0	2.2	1.27 bc	0.09	0.4	0.4	0.28 a	0.06
*S. daphnoides*	3.2	2.2	1.34 ab	0.15	0.30	0.25	0.24 a	0.09
*S. fragilis*	6	6	1.82 a	0.09	0.05	0.15	0.05 a	0.06
*S. hastata*	1.8	2.2	0.97 ab	0.30	1	3	0.55 a	0.16
*S. lapponum*	1.3	0.6	0.79 bc	0.25	3.1	0.4	1.40 b	0.16
*S. purpurea*	8	17	1.56 ac	0.09	0.3	0.8	0.21 a	0.06
*S. viminalis*	2.9	2.9	1.20 ab	0.25	0.3	0.5	0.21 a	0.16
*S. × sepulcralis*	3.3	2.4	1.35 ab	0.25	0.10	0.25	0.08 a	0.16

Different letters indicate interspecific significant differences in content (*p* < 0.05). SD—standard deviation; R^2^—regression coefficient; SE—standard error of regression coefficient.

**Table 4:** Summary of the overall effects from linear mixed models and type II ANOVA of the variables species, year, month, and sex on CAD, CAG, and flavan-3-ol content. R^2^ in general are log-transformed concentration estimates in mg/g DW. R^2^ of year relates to 2018 as reference, R^2^ of month refers to May as reference, and R^2^ of sex refers to females as reference.

	CAD				CAG				Flavan-3-ol			
	R^2^	SE	χ^2^	d.f.	R^2^	SE	χ^2^	d.f.	R^2^	SE	χ^2^	d.f.
**Species**	NA	NA	2015.263 ***	12	NA	NA	368.879 ***	12	NA	NA	2443.197 ***	12
**Year**	0.11 **	0.03	10.719 **	1	−0.005	0.012	0.180	1	−0.18 **	0.06	10.492 **	1
**Month**	−0.047 ***	0.011	17.330 ***	1	0.031 ***	0.005	59.413 ***	1	0.039 *	0.020	3.951 *	1
**Sex**	0.02	0.09	0.124	2	0.006	0.014	0.976	2	0.07	0.11	0.824	2

R^2^—regression coefficient; SE—standard error of regression coefficient; χ^2^—chi-squared value; d.f.—degree of freedom; NA—not detectable. * *p* < 0.05, ** *p* < 0.01, *** *p* < 0.001.

**Table 5:** Summary of the overall effects from linear mixed models and type II ANOVA of the species, year, month, and sex variables on flavanone, flavanonol, flavone, and flavonol contents. R^2^ in general are log-transformed concentration estimates in mg/g DW. R^2^ of year relates to 2018 as reference, R^2^ of month refers to May as reference, and R^2^ of sex refers to females as reference.

	Flavanone				Flavanonol				Flavone				Flavonol			
	R^2^	SE	χ^2^	d.f.	R^2^	SE	χ^2^	d.f.	R^2^	SE	χ^2^	d.f.	R^2^	SE	χ^2^	d.f.
**Species**	NA	NA	1311.313 ***	12	NA	NA	80.713 ***	12	NA	NA	1366.758 ***	12	NA	NA	285.123 ***	12
**Year**	0.052	0.027	3.823	1	−0.006	0.028	0.041	1	0.10	0.06	3.132	1	0.001	0.020	0.002	1
**Month**	0.033 ***	0.010	11.974 ***	1	0.019	0.010	3.772	1	0.064 **	0.021	9.740 **	1	0.015 *	0.007	4.868 *	1
**Sex**	0.20	0.13	3.100	2	−0.11	0.11	1.440	2	−0.02	0.09	0.065	2	−0.08	0.05	2.939	2

R^2^—regression coefficient; SE—standard error of regression coefficient; χ^2^—chi-squared value; d.f.—degree of freedom; NA—not detectable. * *p* < 0.05, ** *p* < 0.01, *** *p* < 0.001.

**Table 6:** Pearson correlation coefficients (r) among each phenolic compound class were calculated across all investigated species, individuals, sampling months (May–September), and years (2018–2019).

	SAD	CAD	CAG	Flavan-3-ol	Flavanone	Flavanonol	Flavone	Flavonol
**SAD**	1.000	0.092	−0.109 *	−0.361 ***	0.366 ***	0.021	0.049	−0.049
**CAD**	0.092	1.000	−0.052	−0.374 ***	−0.173 ***	−0.022	0.176 ***	0.119 *
**CAG**	−0.109 *	−0.052	1.000	−0.107 *	−0.161 ***	0.223 ***	−0.010	0.352 ***
**Flavan-3-ol**	−0.361 ***	−0.374 ***	−0.107 *	1.000	−0.183 ***	−0.143 **	−0.173 ***	−0.129 **
**Flavanone**	0.366 ***	−0.173 ***	−0.161 ***	−0.183 ***	1.000	−0.030	0.075	−0.118 *
**Flavanonol**	0.021	−0.022	0.223 ***	−0.143 **	−0.030	1.000	0.041	0.566 ***
**Flavone**	0.049	0.176 ***	−0.010	−0.173 ***	0.075	0.041	1.000	0.214 ***
**Flavonol**	−0.049	0.119 *	0.352 ***	−0.129 **	−0.118 *	0.566	0.214 ***	1.000

* *p* < 0.05, ** *p* < 0.01, *** *p* < 0.001.

## Data Availability

Dataset available on request from the authors.

## Supplementary Materials

The following supporting information can be downloaded at https://www.mdpi.com/article/10.3390/plants15111712/s1, Figure S1: Overview of the variation in the caffeic acid derivative (A), coumaryl alcohol glucoside (B), flavan-3-ol (C), flavanone (D), flavanonol (E), flavone (F), and flavonol (G) levels during the growing period across both investigated years (interseasonal variability). Data are shown as mean ± SD. For species represented by a single individual, the mean for each month includes the data across both years for the concerning individual. For species represented by more than one individual, the mean for each month includes the data of each individual across both years.; Figure S2: Climatic data to temperature in the Ecological-Botanical Gardens of the University of Bayreuth (ÖBG Bayreuth), Germany in 2018.; Figure S3: Climatic data to percipitation in the Ecological-Botanical Gardens of the University of Bayreuth (ÖBG Bayreuth), Germany in 2018.; Figure S4: Climatic data to temperature in the Ecological-Botanical Gardens of the University of Bayreuth (ÖBG Bayreuth), Germany in 2019.; Figure S5: Climatic data to percipitation in the Ecological-Botanical Gardens of the University of Bayreuth (ÖBG Bayreuth), Germany in 2019.; Figure S6: A representative photograph of Salix aurita L. showing typical morphological features.; Figure S7: Representative images of *Salix bicolor* EHRH. ex WILLD. (A) Flowering shoots. (B) Leaf morphology.; Figure S8: Representative photograph of Salix caesia L. showing typical morphological features.; Figure S9: A representative photograph of Salix caprea L. showing typical morphological features.; Figure S10: Representative photograph of Salix cinerea L. showing typical morphological features.; Figure S11: A representative photograph of *Salix daphnoides* VILL. showing typical morphological features.; Figure S12: Representative photograph of Salix fragilis L. showing typical morphological features.; Figure S13: Representative images of *Salix hastata* L. (A) Flowering shoots. (B) Leaf morphology.; Figure S14: A representative photograph of Salix lapponum L. showing typical morphological features.; Figure S15: Representative images of *Salix purpurea* L. (A) Flowering shoots. (B) Leaf morphology.; Figure S16: Representative images of *Salix viminalis* L. (A) Flowering shoots. (B) Leaf morphology.; Figure S17: Representative images of *Salix × sepulcralis* SIMONK. (A) Leaf morphology. (B) Flowering shoots.; Table S1: Overview of the investigated species, the respective sex, number of individuals per species, intern identification number, corresponding voucher number, GPS coordinates of the respective location of the individual, and common synonyms [3]. Species identification was verified by Gregor Aas [4,109] and voucher specimens of all investigated individuals were deposited in the herbarium ID UBT/Ecological-Botanical Gardens of the University of Bayreuth (ÖBG Bayreuth), Germany.

## Abbreviations

The following abbreviations are used in this manuscript:

SAD	Salicylic alcohol derivative
CAD	Caffeic acid derivative
CAG	Coumaryl alcohol glucoside
DW	Dry weight
PCA	Principal component analysis
SD	Standard deviation
HSD	Honest significant difference
ACN	Acetonitrile
MeOH	Methanol
r	Pearson correlation coefficients
