# Supplementary material for: Investigation of the Phenolic Profile and Chemotaxonomical Screening of Twelve Salix Species During Growing Season
Source: Plants (Basel). 2026 Jun 1;15(11):1712. doi: 10.3390/plants15111712 (PMC13259341; doi:10.3390/plants15111712)

Figure S1. Overview of the variation in the caffeic acid derivative (A), coumaryl alcohol glucoside (B), flavan-3-ol (C), flavanone (D), flavanonol (E), flavone (F), and flavonol (G) levels during the growing period across both investigated years (interseasonal variability). Data are shown as mean ± SD. For species represented by a single individual, the mean for each month includes the data across both years for the concerning individual. For species represented by more than one individual, the mean for each month includes the data of each individual across both years.

(A)

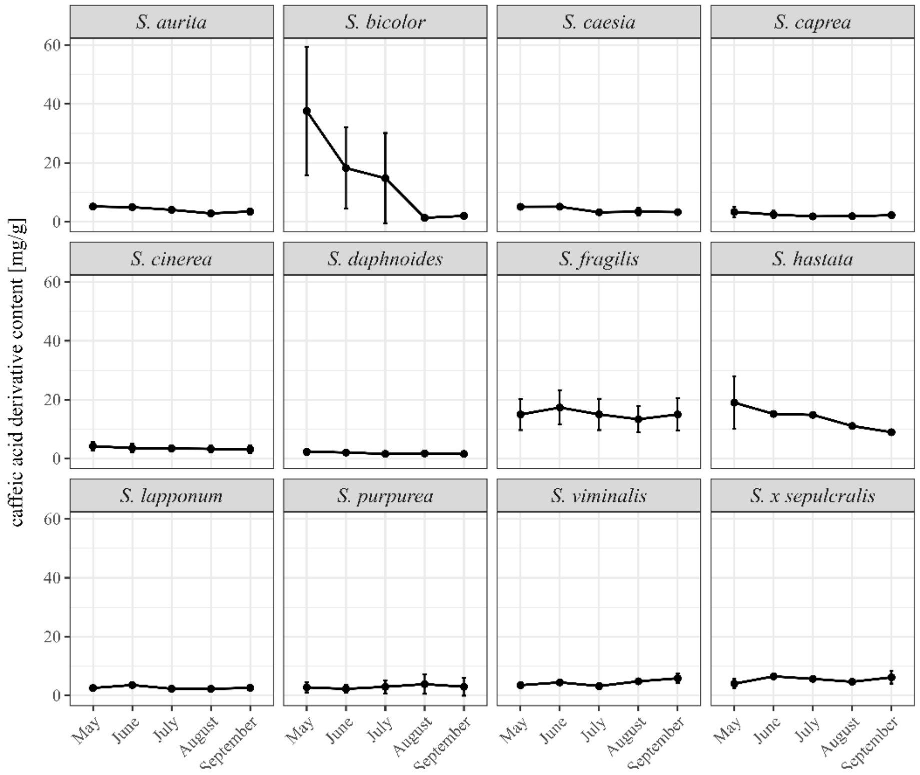

(B)

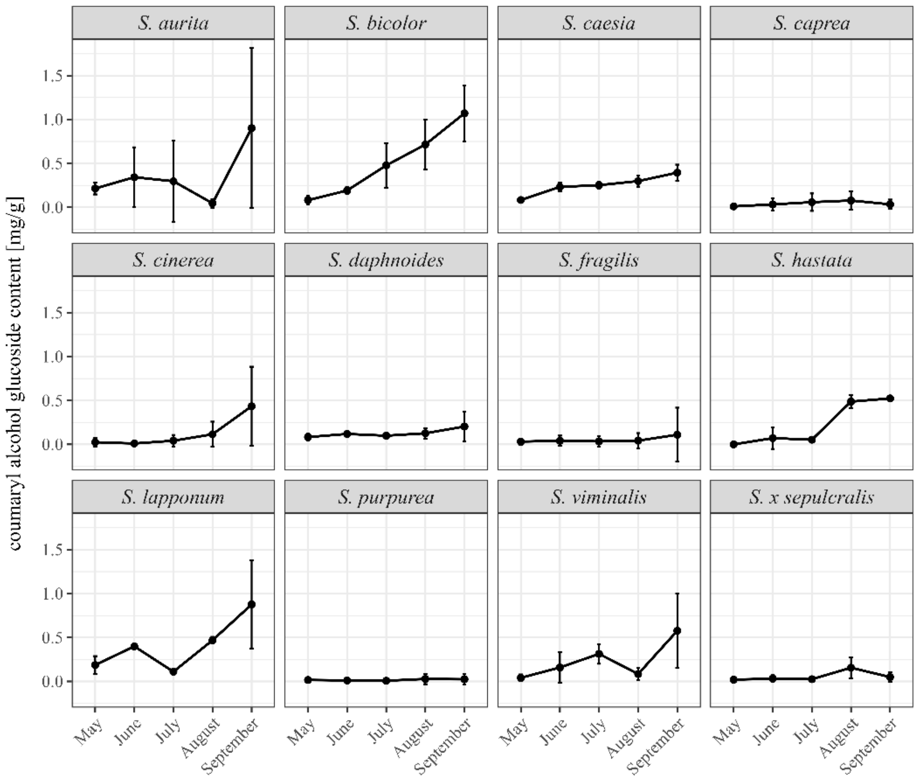

(C)

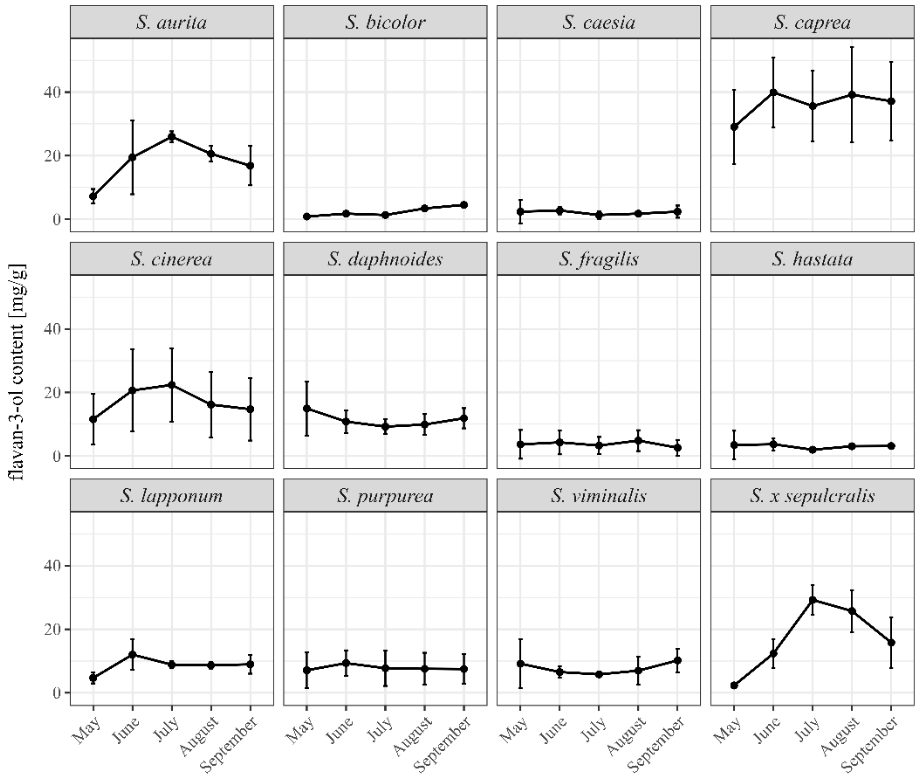
S. aurita
S. bicolor
S. caesia
S. caprea
S. cinerea
S. daphnoides
S. fragilis
S. hastata
S. lapponum
S. purpurea
S. viminalis
S. x sepulcralis
flavan-3-ol content [mg/g]
0
20
40
May
June
July
August
September

(D)

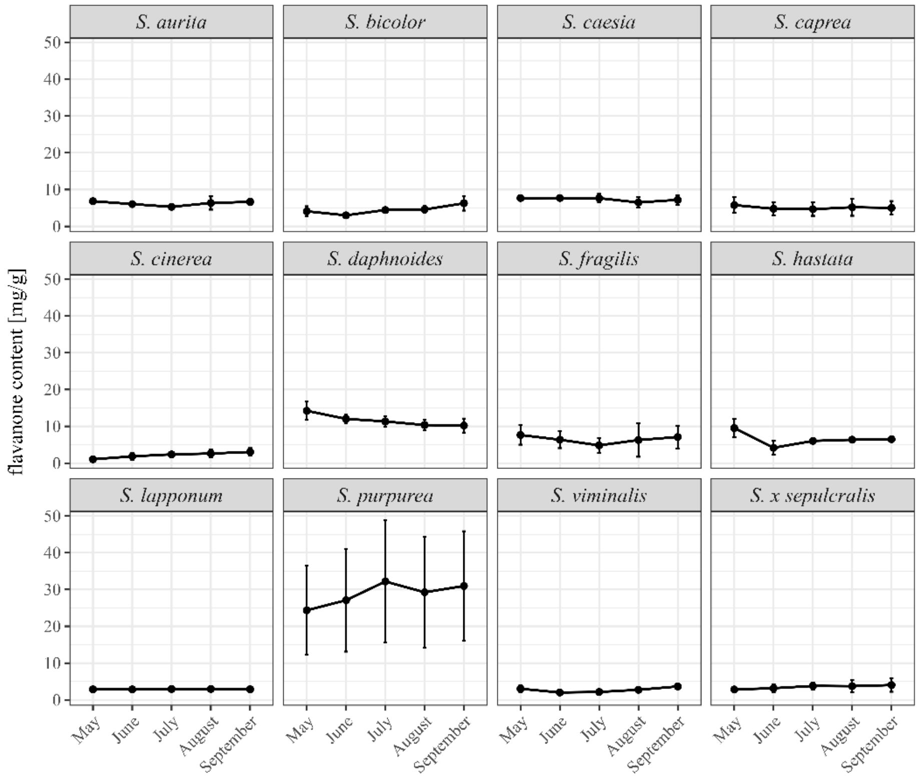
S. aurita
S. bicolor
S. caesia
S. caprea
S. cinerea
S. daphnoides
S. fragilis
S. hastata
S. lapponum
S. purpurea
S. viminalis
S. x sepulcralis
flavanone content [mg/g]
0
10
20
30
40
50
May
June
July
August
September

(E)

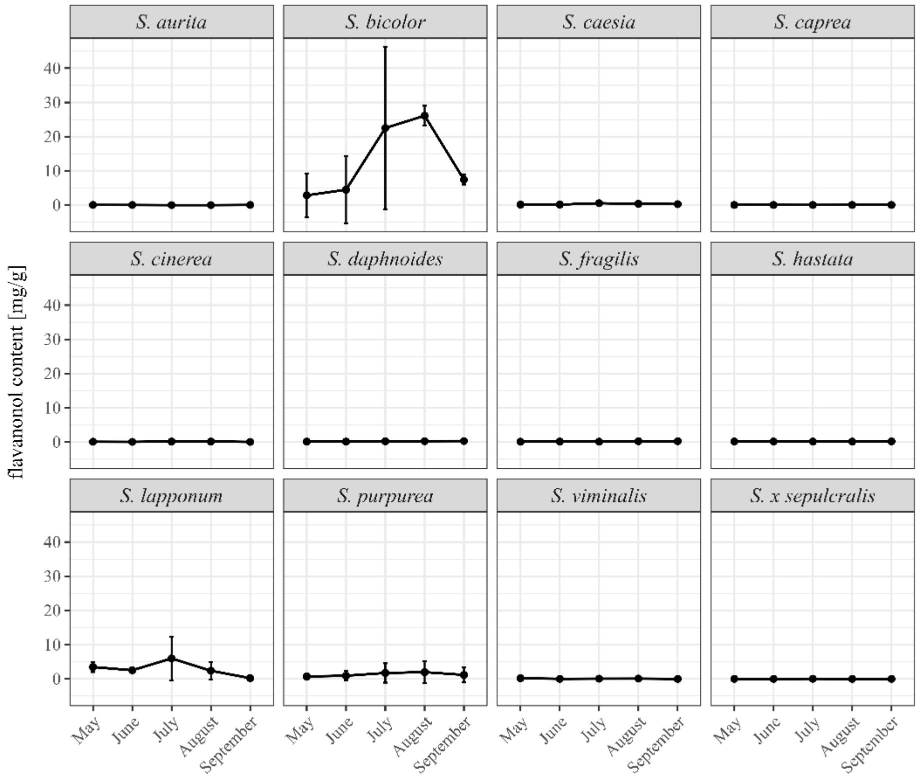
S. aurita
S. bicolor
S. caesia
S. caprea
S. cinerea
S. daphnoides
S. fragilis
S. hastata
S. lapponum
S. purpurea
S. viminalis
S. x sepulcralis
flavanonol content [mg/g]
0
10
20
30
40
May
June
July
August
September

(F)

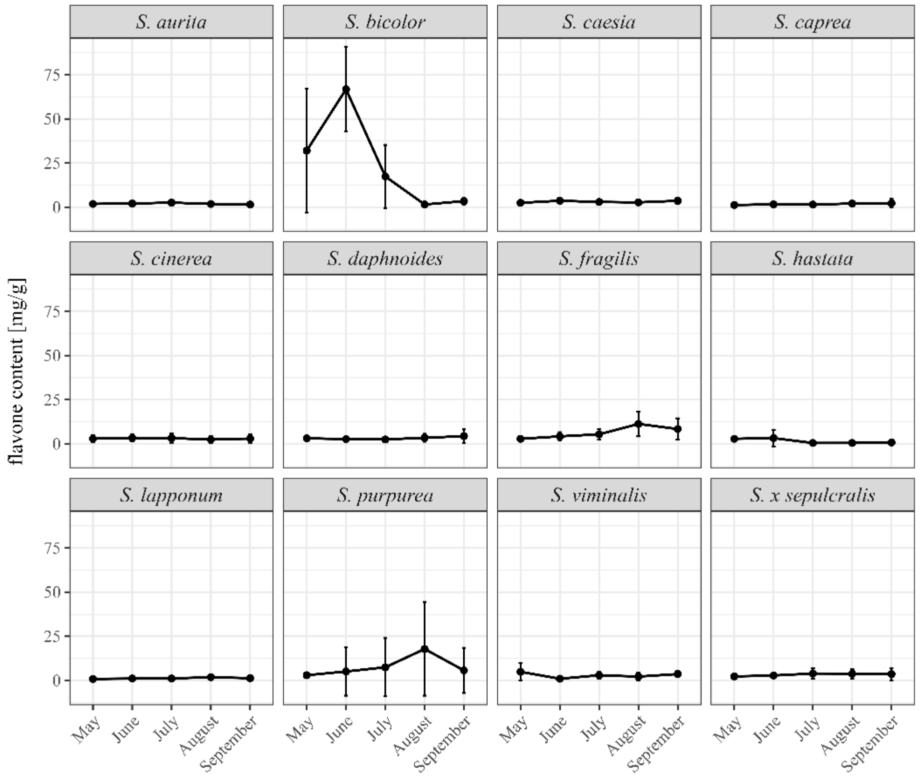
S. aurita
S. bicolor
S. caesia
S. caprea
S. cinerea
S. daphnoides
S. fragilis
S. hastata
S. lapponum
S. purpurea
S. viminalis
S. x sepulcralis
flavone content [mg/g]
0
25
50
75
May
June
July
August
September

(G)

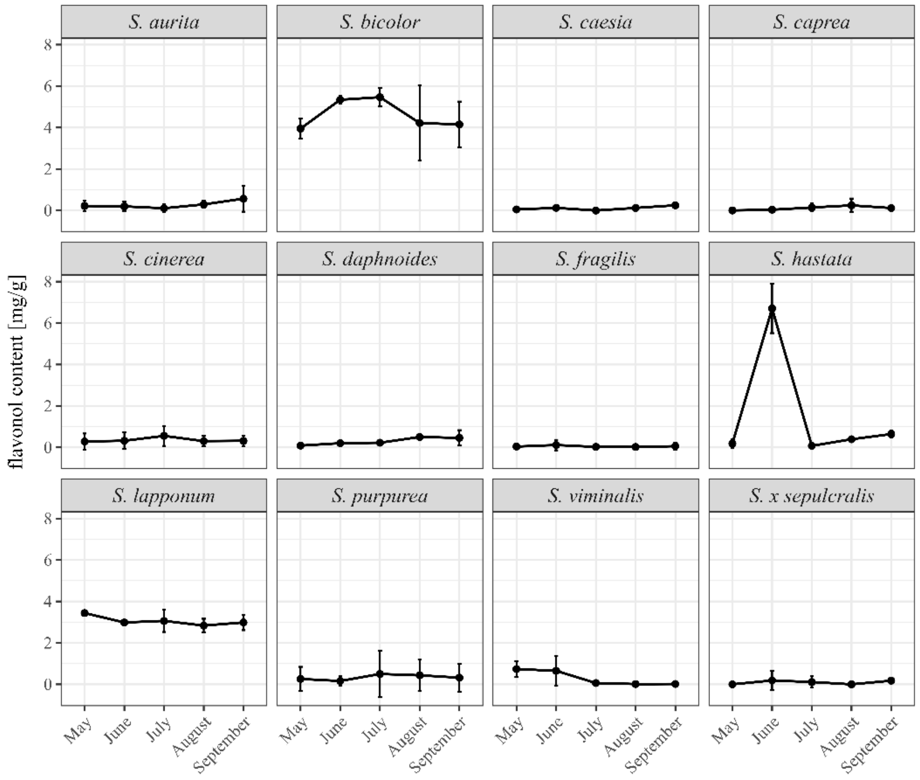
S. aurita
S. bicolor
S. caesia
S. caprea
S. cinerea
S. daphnoides
S. fragilis
S. hastata
S. lapponum
S. purpurea
S. viminalis
S. x sepulcralis
flavonol content [mg/g]
0
2
4
6
8
May
June
July
August
September

Table S1. Overview of the investigated species, the respective sex, number of individuals per species, intern identification number, corresponding voucher number, GPS coordinates of the respective location of the individual, and common synonyms [3]. Species identification was verified by Gregor Aas [4,109] and voucher specimens of all investigated individuals were deposited in the herbarium ID UBT/Ecological-Botanical Gardens of the University of Bayreuth (ÖBG Bayreuth), Germany.

Species	Sex	Number of individuals	Identification Number	Voucher Number	GPS coordinates	Synonym
Salix aurita L.	female	1	22_Saur_f_BT	48069	N49 55.398<br>E11 34.970	
Salix bicolor EHRH. ex WILLD.	female	1	19_Sbic_f_BT	48063	N49 55.518<br>E11 34.963	*Salix phylicifolia* L.,<br>*Salix schraderiana* WILLD.
Salix caesia VILL.	female	1	21_Scae_f_BT	48065	N49 55.518<br>E11 34.963	
Salix caprea L.	female	4	26_Scap_f_BT	48073	N49 55.393<br>E11 35.085	
			27_Scap_f_BT	48074	N49 55.386<br>E11 35.105	
			28_Scap_f_BT	48075	N49 55.366<br>E11 34.940	
			29_Scap_f_BT	48076	N49 55.487<br>E11 34.980	
	male	4	30_Scap_m_BT	48077	N49 55.393<br>E11 35.085	
			31_Scap_m_BT	48078	N49 55.378<br>E11 34.993	
			32_Scap_m_BT	48079	N49 55.487<br>E11 34.980	
			33_Scap_m_BT	48080	N49 55.518<br>E11 34.963	
Salix cinerea L.	female	4	37_Scin_f_BT	48085	N49 55.398<br>E11 34.970	
			38_Scin_f_BT	48086	N49 55.387<br>E11 34.956	

			39_Scin_f_BT	48087	N49 55.366<br>E11 34.940	
			40_Scin_f_BT	48088	N49 55.366<br>E11 34.940	
	male	4	08_Scin_m_BT	48081	N49 55.484<br>E11 35.036	
			34_Scin_m_BT	48082	N49 55.484<br>E11 35.036	
			35_Scin_m_BT	48083	N49 55.518<br>E11 34.963	
			36_Scin_m_BT	48084	N49 55.398<br>E11 34.970	
Salix daphnoides VILL.	female	1	02_Sdap_f_BT	48066	N49 55.518<br>E11 34.963	
	male	2	03_Sdap_m_BT	48067	N49 55.396<br>E11 35.105	
			16_Sdap_m_BT	48068	N49 55.401<br>E11 35.013	
Salix fragilis L.	female	4	18_Sfra_f_BT	48090	N49 55.438<br>E11 35.127	*Salix euxina* I. V. BELYAEVA
			41_Sfra_f_BT	48089	N49 55.824<br>E11 35.017	
			42_Sfra_f_BT	48091	N49 55.320<br>E11 34.838	
			43_Sfra_f_BT	48092	N49 55.320<br>E11 34.838	
	male	4	04_Sfra_m_BT	48093	N49 55.435<br>E11 35.089	
			17_Sfra_m_BT	48094	N49 55.435<br>E11 35.089	
			44_Sfra_m_BT	48095	N49 55.336<br>E11 34.957	

			45_Sfra_m_BT	48096	N49 55.336<br>E11 34.957	
Salix hastata L.	female	1	20_Shas_f_BT	48064	N49 55.518<br>E11 34.963	
Salix lapponum L.	female	1	24_Slap_f_BT	48071	N49 55.396<br>E11 35.105	
Salix purpurea L.	female	5	14_Spur_f_BT	48098	N49 55.518<br>E11 34.963	
			15_Spur_f_BT	48099	N49 55.430<br>E11 35.229	
			46_Spur_f_BT	48097	N49 55.295<br>E11 34.908	
			47_Spur_f_BT	48100	N49 55.068<br>E11 35.431	
			50_Spur_f_BT	48104	N49 55.673<br>E11 35.213	
	male	3	01_Spur_m_BT	48103	N49 55.518<br>E11 34.963	
			48_Spur_m_BT	48101	N49 55.387<br>E11 34.956	
			49_Spur_m_BT	48102	N49 55.068<br>E11 35.431	
Salix viminalis L.	male	1	23_Svim_m_BT	48070	N49 55.408<br>E11 35.025	
Salix × *sepulcralis* SIMONK.	monoecious	1	25_Sxse_mf_BT	48072	N49 55.422<br>E11 34.990	*Salix alba* var. *vitellina* × *Salix babylonica*

Figure S2. Climatic data to temperature in the Ecological-Botanical Gardens of the University of Bayreuth (ÖBG Bayreuth), Germany in 2018.

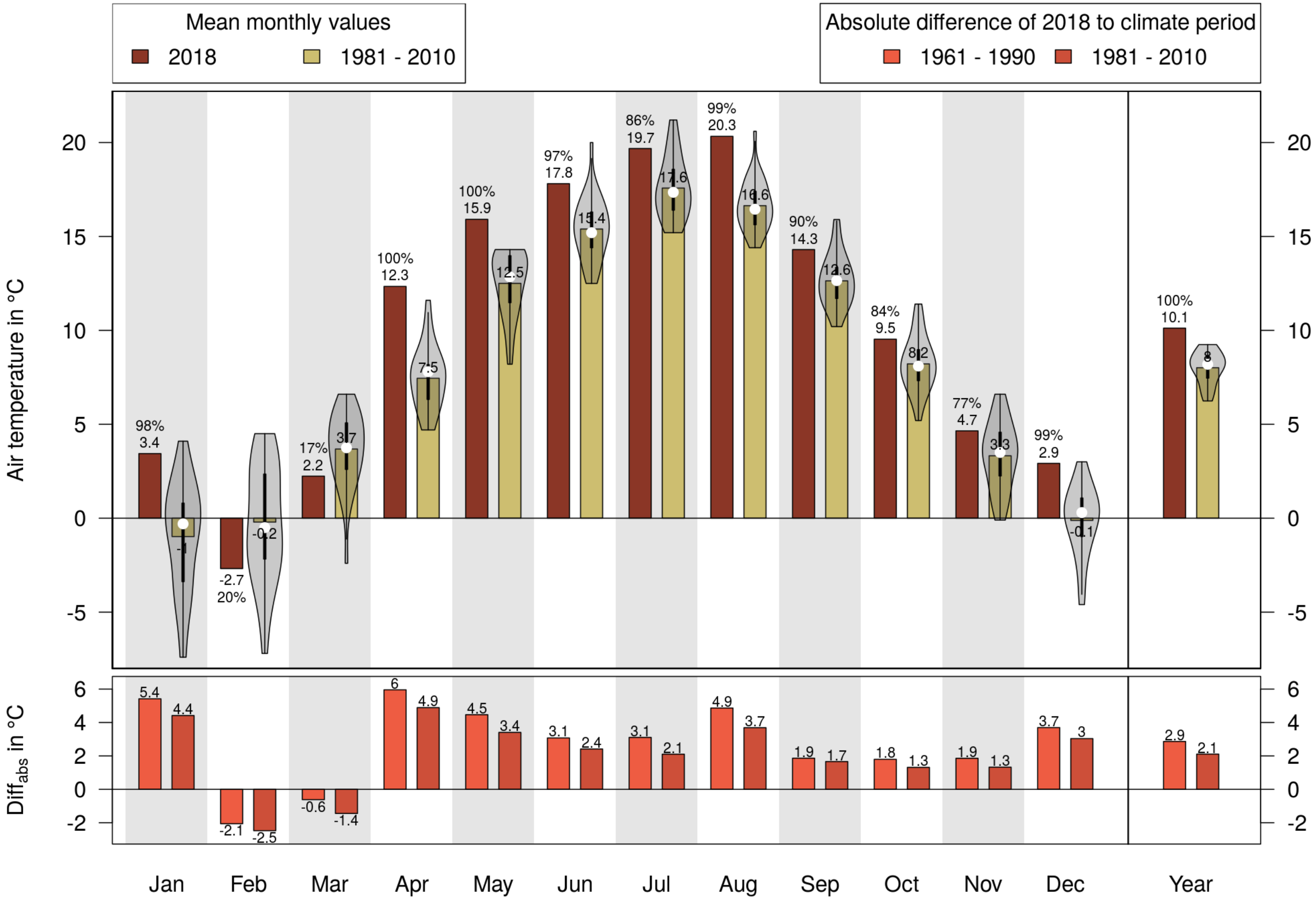

Figure S3. Climatic data to percipitation in the Ecological-Botanical Gardens of the University of Bayreuth (ÖBG Bayreuth), Germany in 2018.

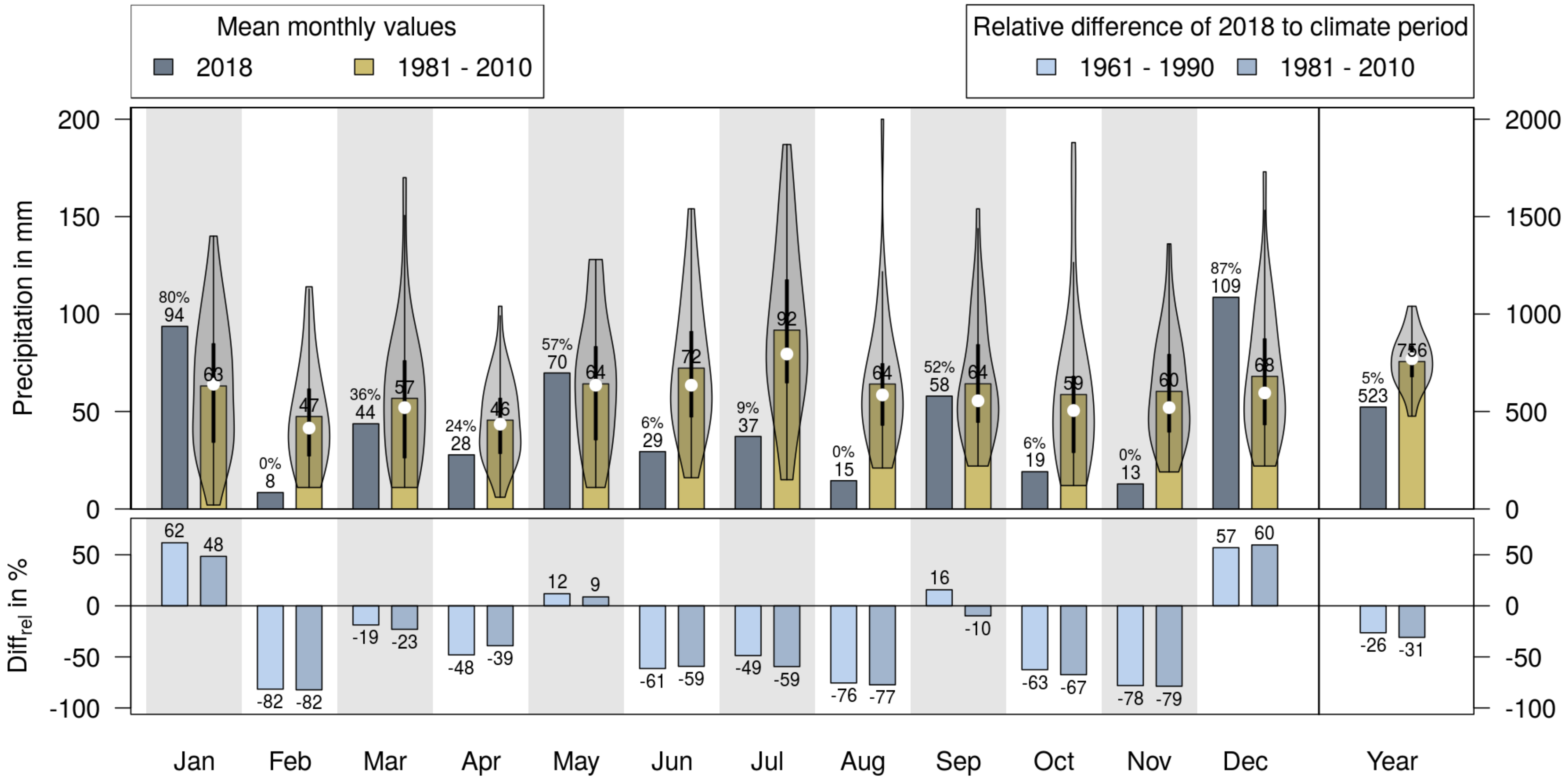

Figure S4. Climatic data to temperature in the Ecological-Botanical Gardens of the University of Bayreuth (ÖBG Bayreuth), Germany in 2019.

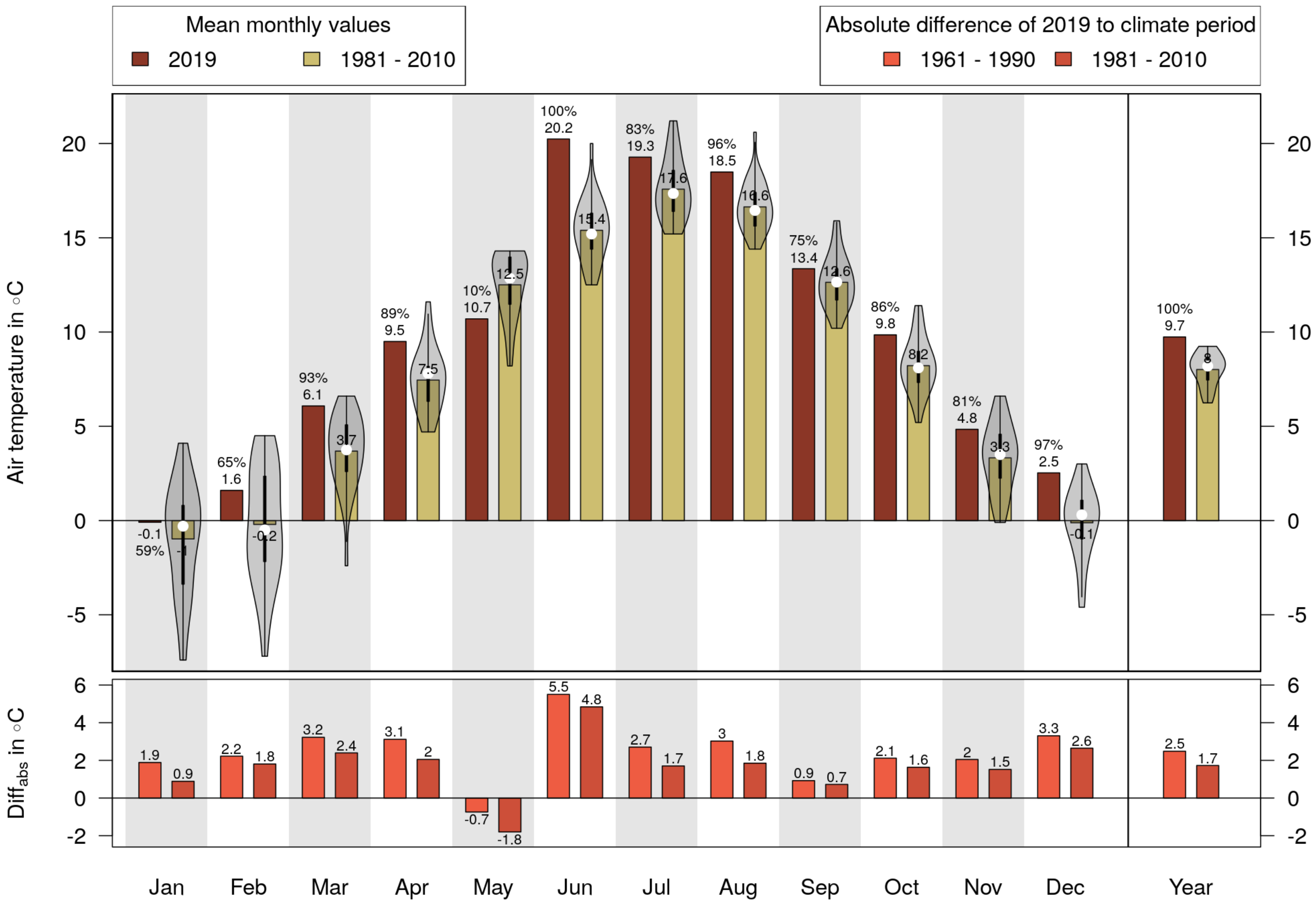

Figure S5. Climatic data to percipitation in the Ecological-Botanical Gardens of the University of Bayreuth (ÖBG Bayreuth), Germany in 2019.

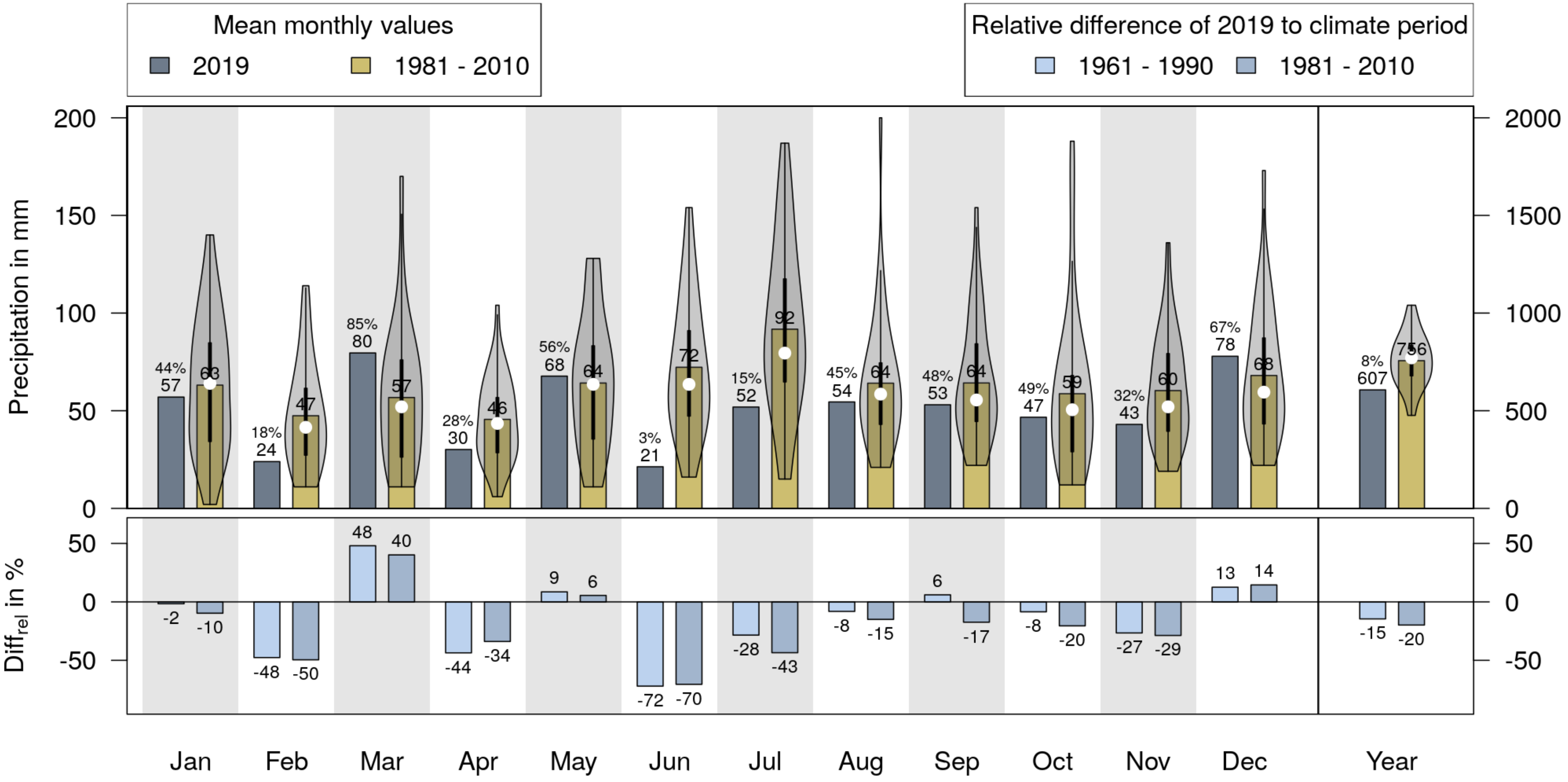

Figure S6. A representative photograph of *Salix aurita* L. showing typical morphological features.

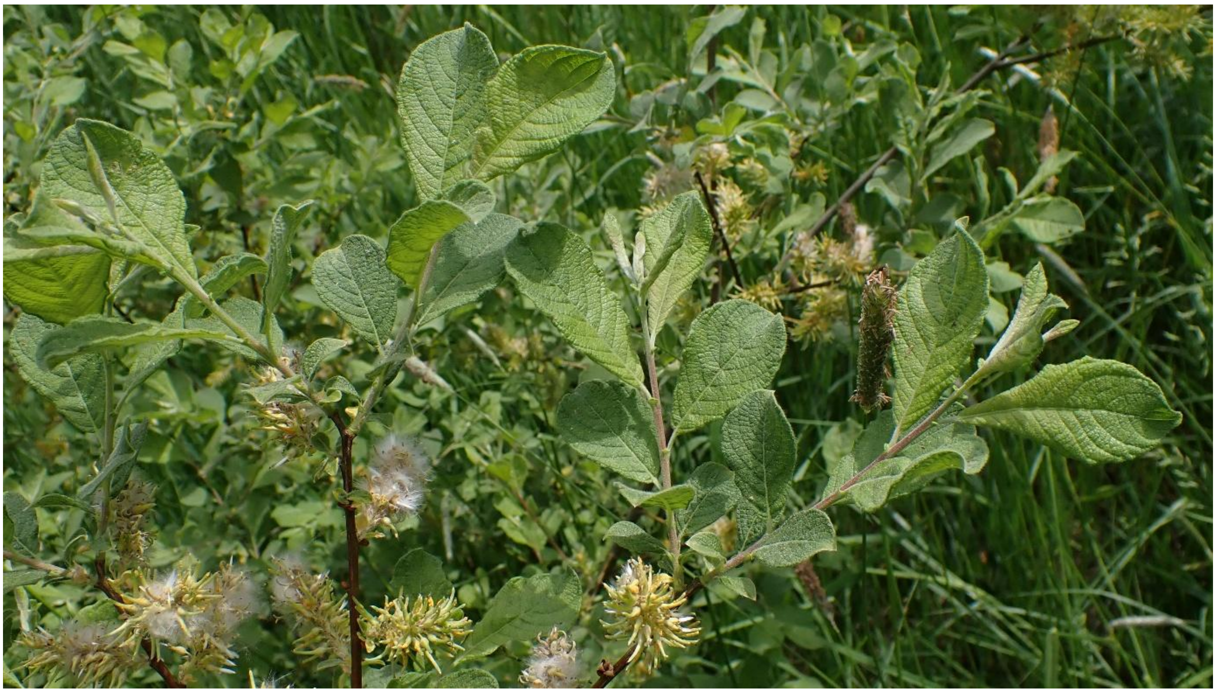

Figure S7. Representative images of *Salix bicolor* EHRH. ex WILLD.. (A) Flowering shoots. (B) Leaf morphology.

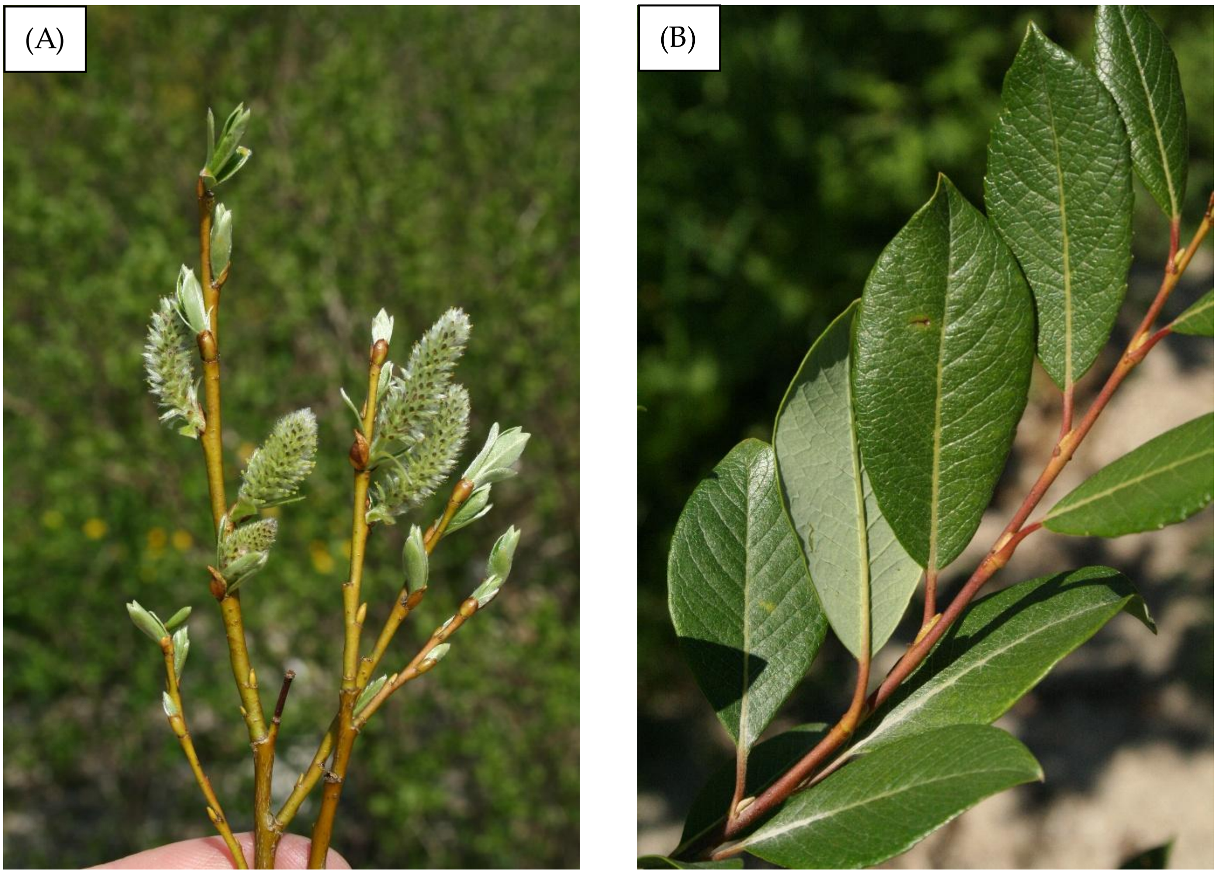

Figure S8. Representative photograph of *Salix caesia* L. showing typical morphological features.

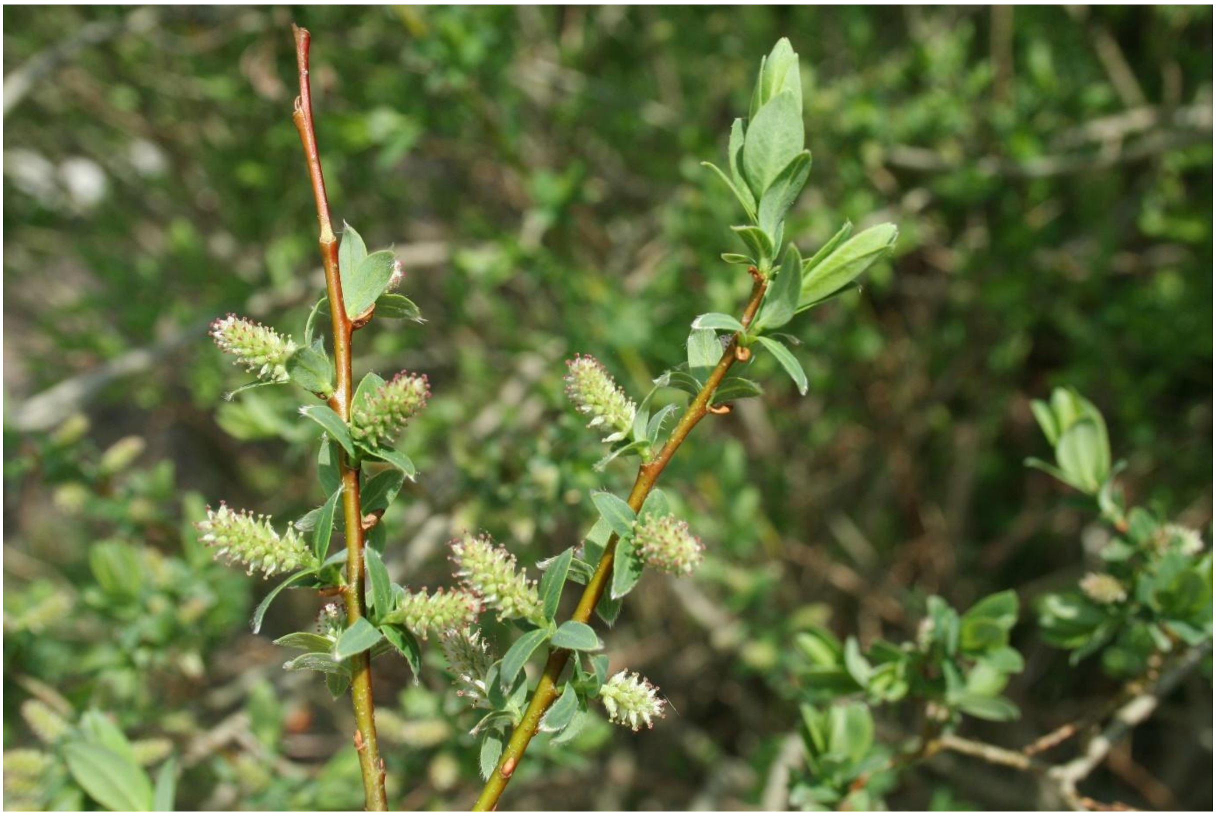

Figure S9. A representative photograph of *Salix caprea* L. showing typical morphological features.

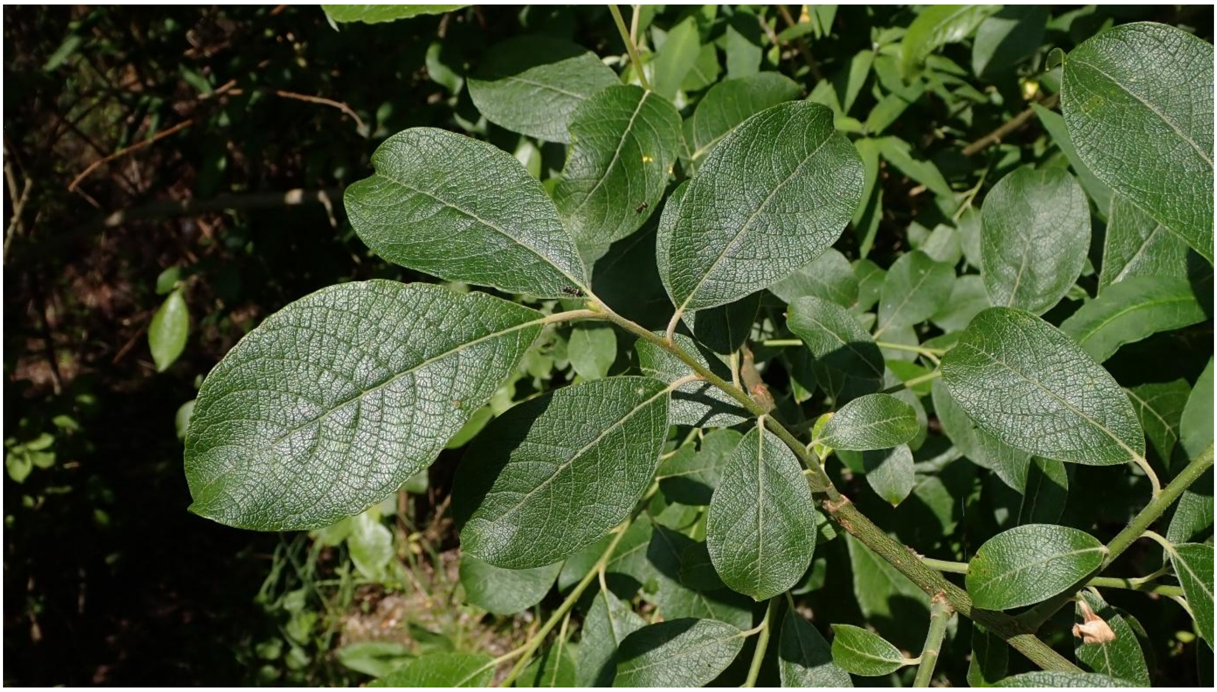

Figure S10. Representative photograph of *Salix cinerea* L. showing typical morphological features.

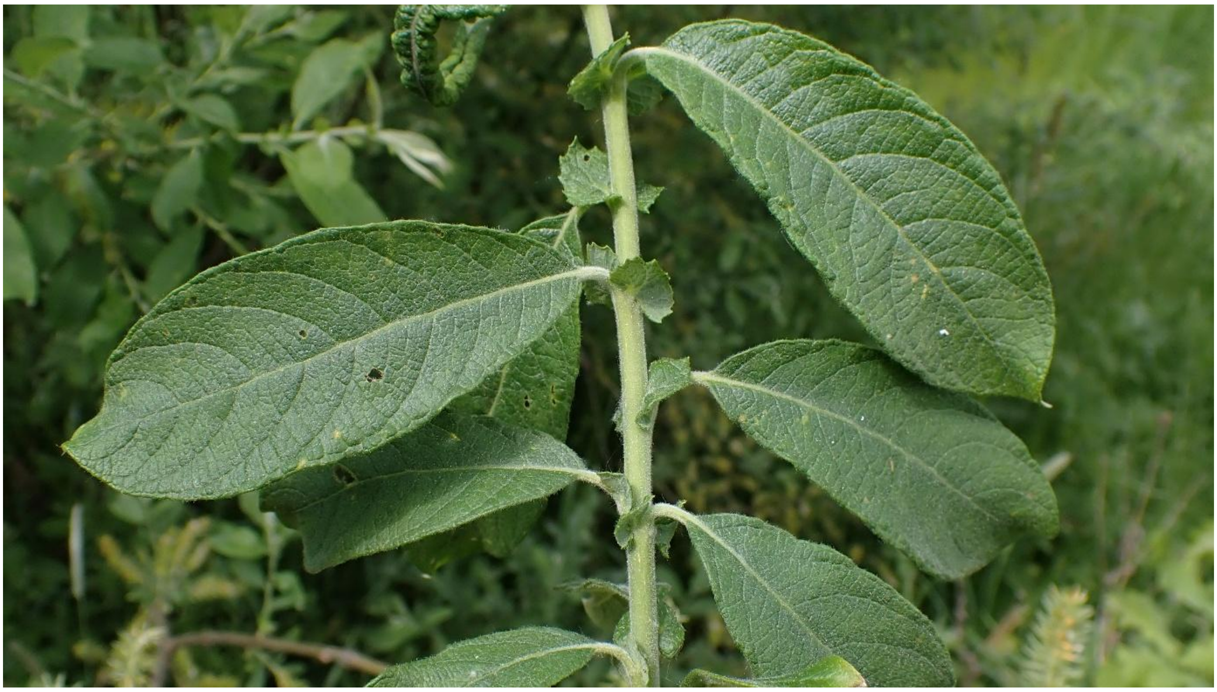

Figure S11. A representative photograph of *Salix daphnoides* VILL. showing typical morphological features.

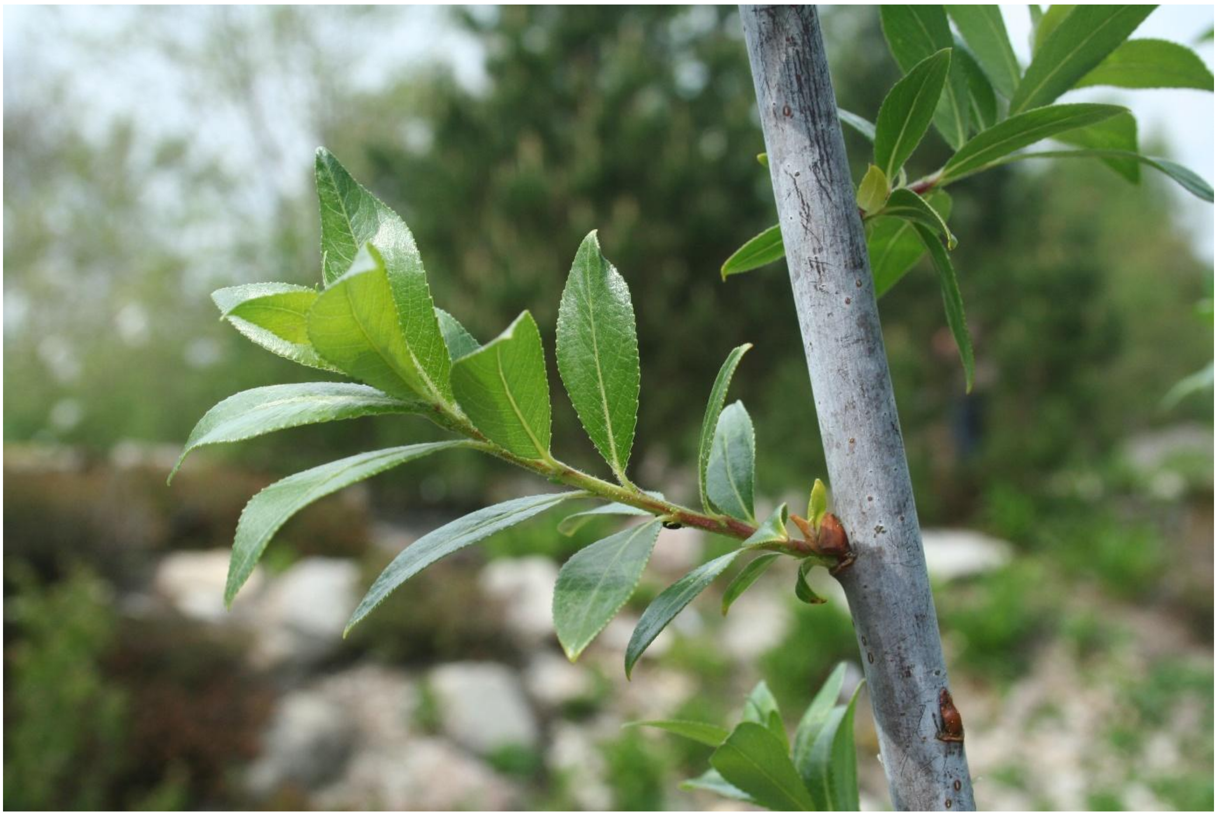

Figure S12. Representative photograph of *Salix fragilis* L. showing typical morphological features.

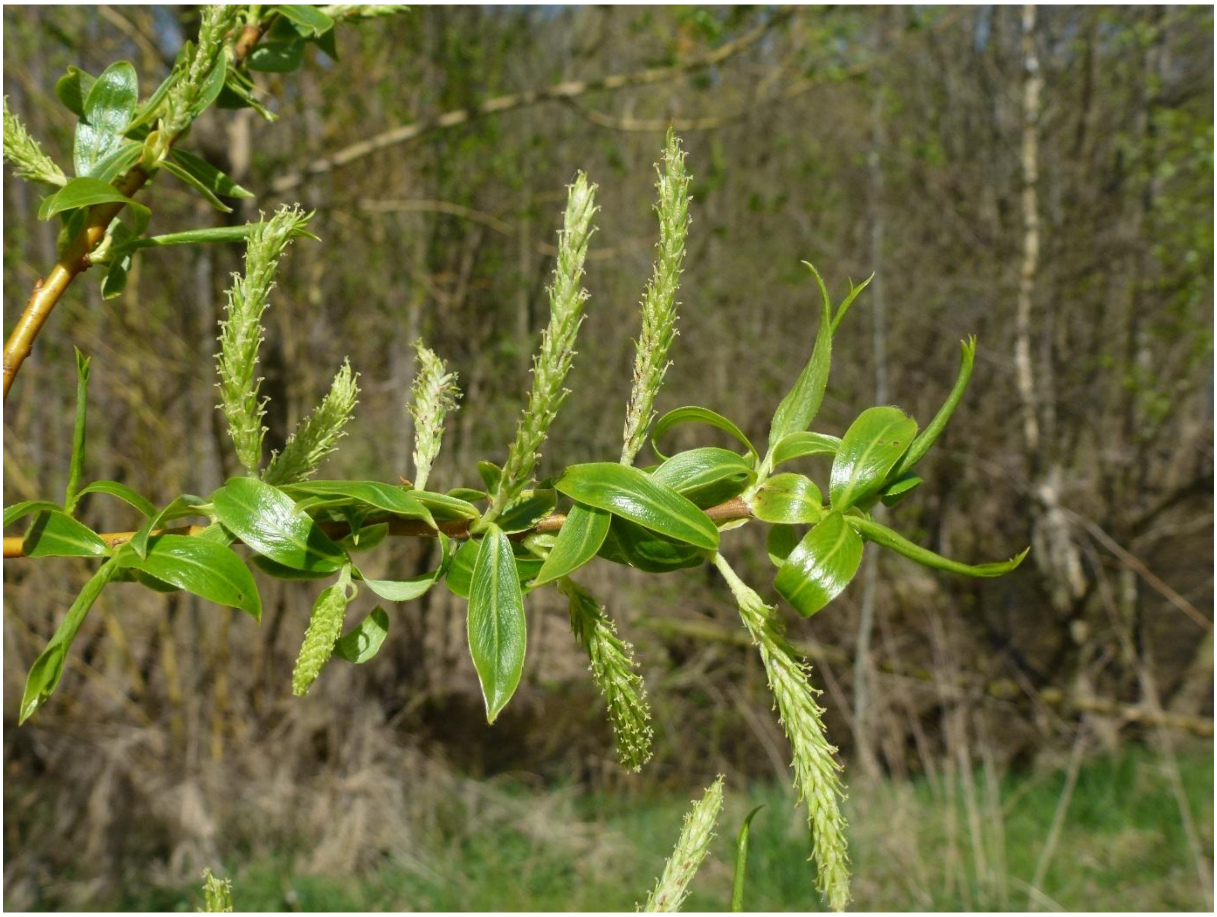

Figure S13. Representative images of *Salix hastata* L.. (A) Flowering shoots. (B) Leaf morphology.

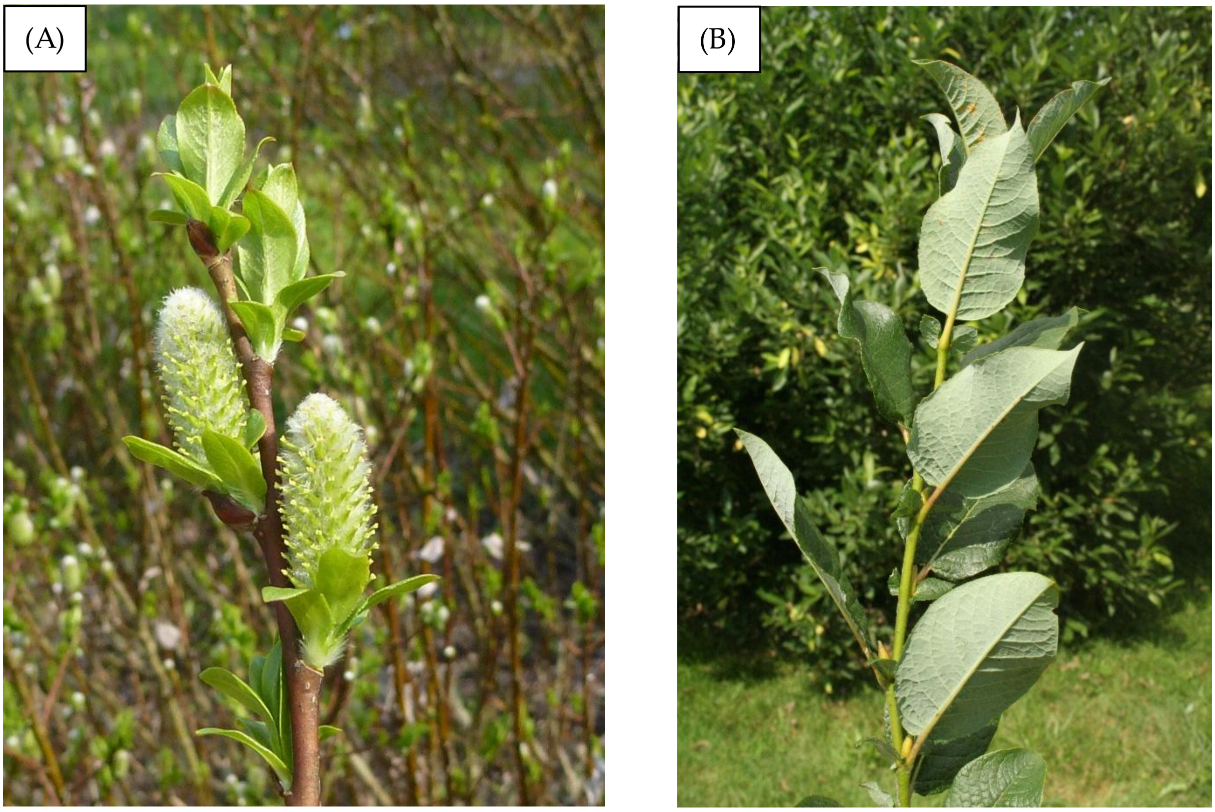

Figure S14. A representative photograph of *Salix lapponum* L. showing typical morphological features.

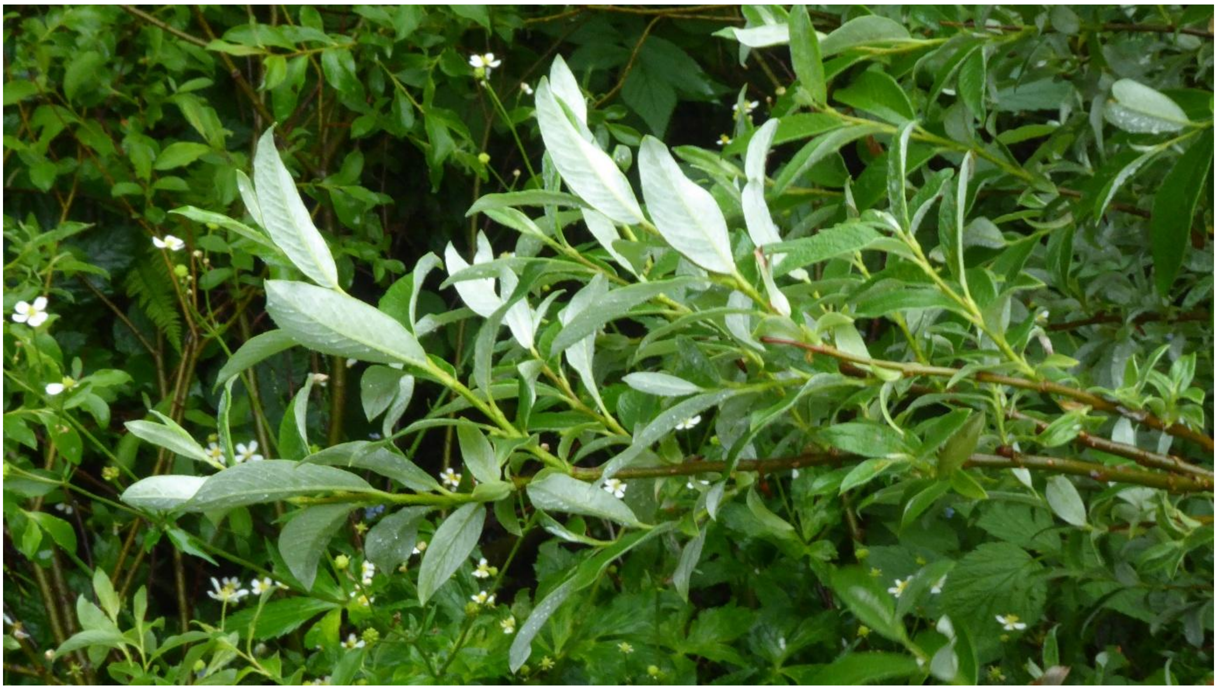

Figure S15. Representative images of *Salix purpurea* L.. (A) Flowering shoots. (B) Leaf morphology.

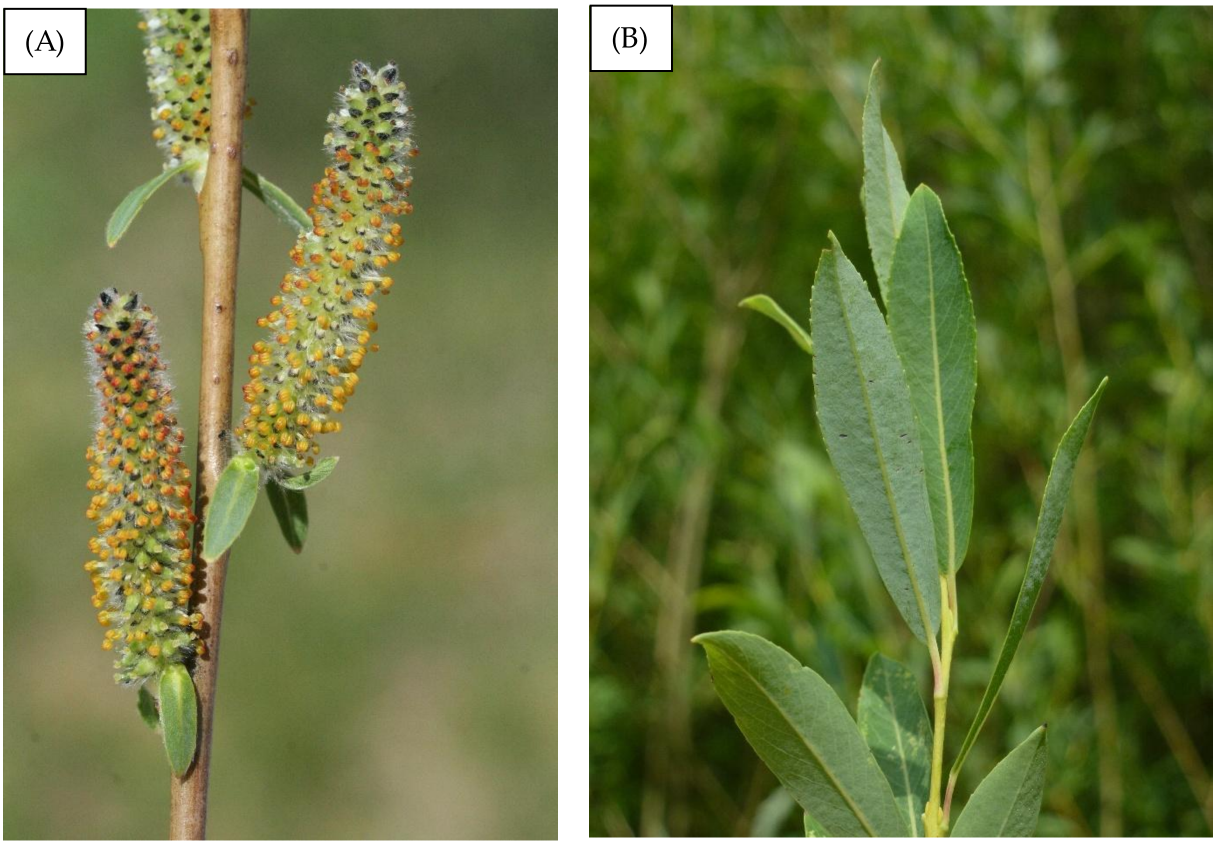

Figure S16. Representative images of *Salix viminalis* L.. (A) Flowering shoots. (B) Leaf morphology.

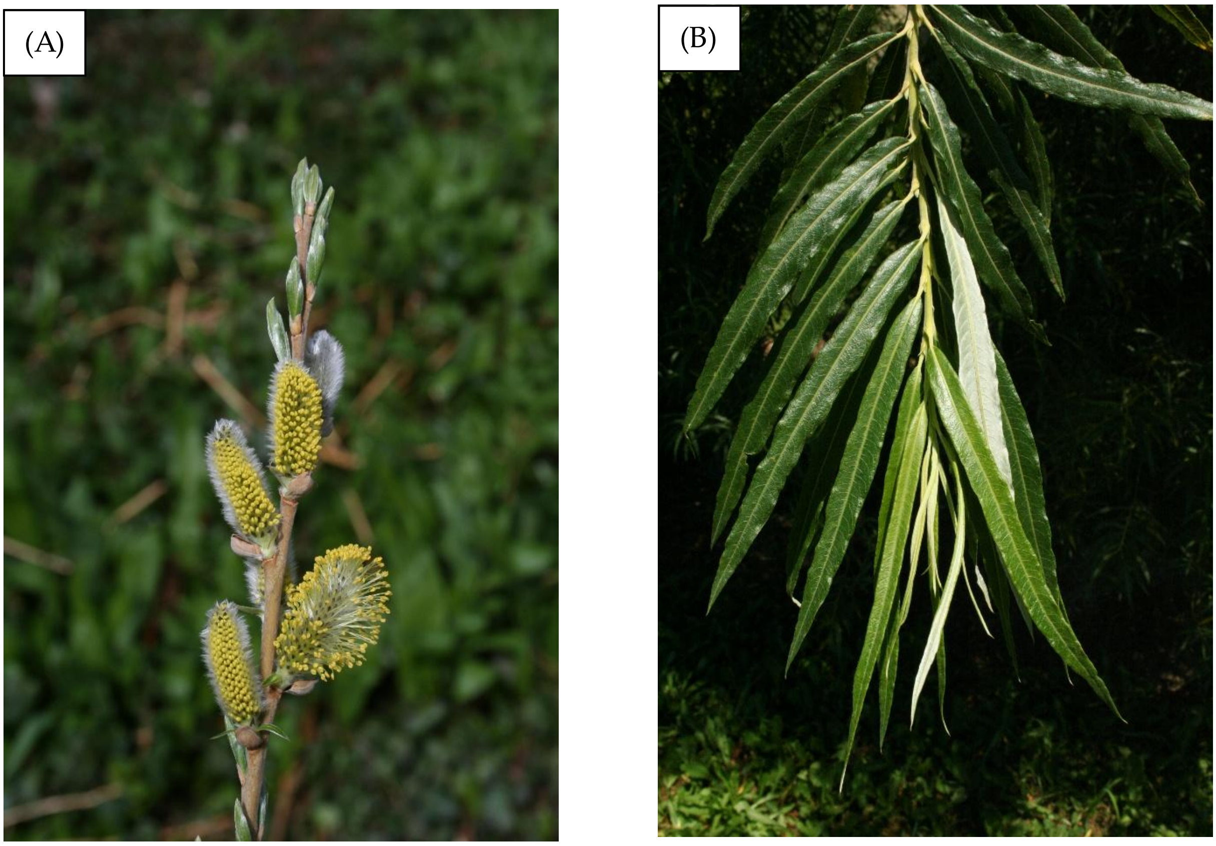

Figure S17. Representative images of *Salix* × *sepulcralis* SIMONK.. (A) Leaf morphology. (B) Flowering shoots.

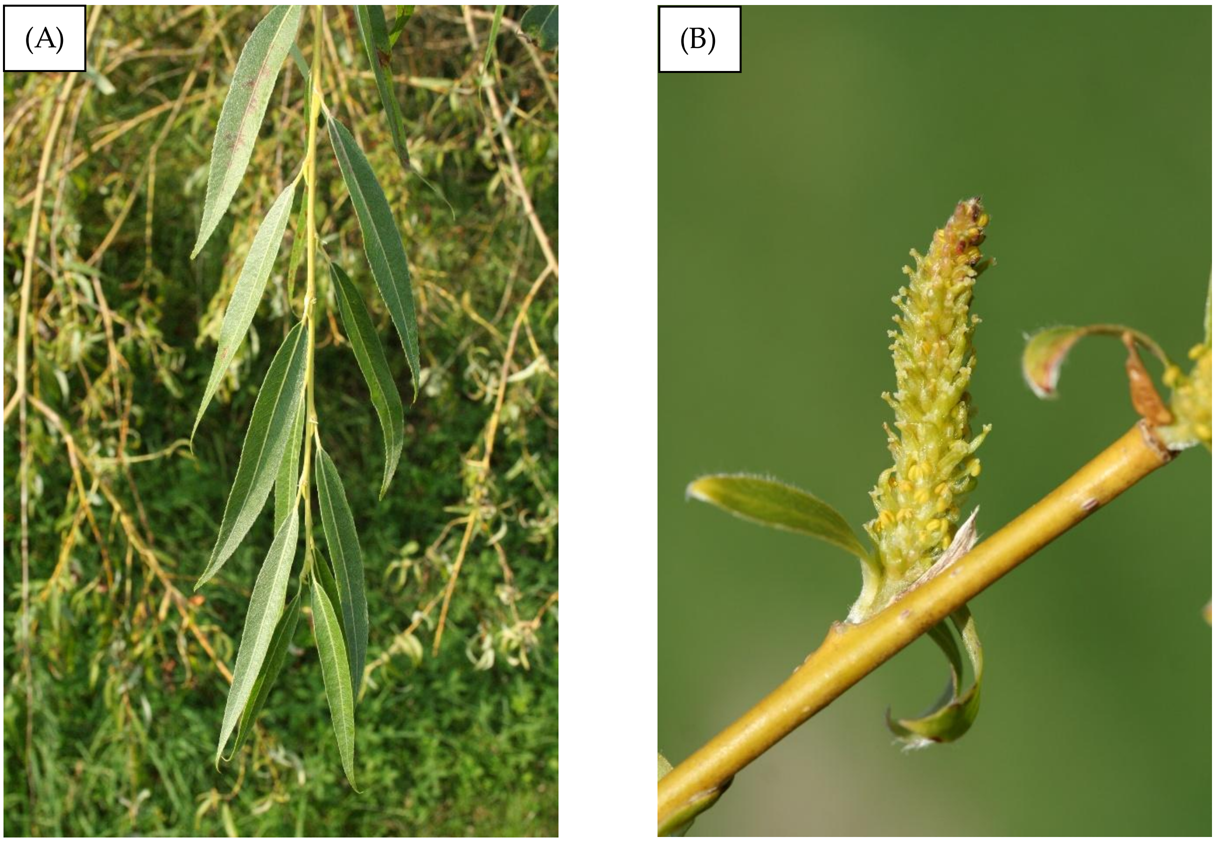

Supplement: Supplementary file 1 [file plants-15-01712-s001.zip › plants-4236878-supplementary.pdf]
